# 3,4-Bis-*O*-propargyl-1,2:5,6-di-*O*-iso­propyl­idene-d-mannitol: a study of multiple weak hydrogen bonds in the solid state

**DOI:** 10.1107/S205322962200897X

**Published:** 2022-10-11

**Authors:** Adnan I. Mohammed, Mohan M. Bhadbhade, Roger W. Read

**Affiliations:** a Department of Chemistry, College of Science, University of Kerbala, Karbala, Iraq; b Mark Wainwright Analytical Centre, The University of New South Wales (UNSW), Sydney, NSW 2052, Australia; c School of Chemistry, The University of New South Wales (UNSW), Sydney, NSW 2052, Australia; The University of Western Australia, Australia

**Keywords:** chemical crystallography, weak hydrogen bonds, noncovalent inter­actions, crystal structure, crystal engineering, supra­molecular chemistry, CSD

## Abstract

The title homochiral com­pound, C_18_H_26_O_6_, **1**, was examined by single-crystal X-ray crystallography in order to understand its potential as a synthetic building block, particularly in inter- and intra­molecular cyclo­condensation reactions. It has also proven to be an excellent model for understanding multiple weak donor–acceptor *D*—H⋯*A* inter­actions involving terminal acetyl­enes as donors and as acceptors. The asymmetric unit of **1** com­prises three almost identical independent mol­ecules, each with the mannitol 2*R*,3*R*,4*R*,5*R* con­figuration and different con­formations. Like independent mol­ecules align in strands through acetyl­enic donor C—H⋯O contacts with equivalent dioxolanyl acceptor groups. Two of the strands are aligned unidirectionally, in parallel, while the third strand aligns perpendicular to the first two, to give inter­woven layers in the supra­molecular structure. A detailed study of the inter­digitation of the second propargyl group from each independent mol­ecule between strands, and of other short inter­strand C—H⋯O contacts, provides new insight into the application of weak hydrogen-bond theory within the context of a con­formationally flexible symmetrical mol­ecule. Analyses of the Cambridge Structural Database using Crystal Packing Features and *ConQuest* search motifs support the importance of the *D*⋯*A* distance parameter, demonstrate the different influences of donor and acceptor types, and reveal the inter­play between H⋯*A* and *D*⋯*A* contributions in different contact types.

## Abstract 2

Primary weak hydrogen bonds in each of three independent mol­ecules in 3,4-bis-*O*-propargyl-1,2:5,6-di-*O*-iso­propyl­idene-d-mannitol are elucidated by single-crystal X-ray diffraction and their importance in mol­ecular con­formation and crystal packing determined. Parallel searches of the Cambridge Structural Database using motifs based on donor and acceptor propargyl *D*—H⋯*A* contacts reveal distance and angle dependencies that are especially sensitive to acceptor type, so, together, these findings will be beneficial in applications for the mol­ecule and its congeners as anion binders and precursors for the synthesis of macrocycles.

## Introduction

Propargyl groups are small and can serve as protection for alcohols that can be selectively removed in the presence of acetonides (1,3-dioxolanyls), methoxymethyl (MOM), benzyl and *tert*-butyldimethylsilyl (TBS) ethers (Manabe *et al.*, 2008 ▸; Rambabu *et al.*, 2013 ▸), and provide subtle enhancement of di­astereoselectivity in the synthesis of β-manno­pyran­osylated disaccharides (Crich *et al.*, 2006 ▸). Proparg­yloxy groups can also undergo a variety of useful transformations in their own right. One of us recently described the synthesis of 3,4-bis-*O*-propargyl-1,2:5,6-di-*O*-iso­propyl­idene-d-mannitol, **1**, and its use in Cu^I^-catalyzed dipolar cyclo­addition reactions with *n*-alkyl azides to generate model com­pounds for potential new gemini surfactants (Mohammed *et al.*, 2012 ▸), and extended this study in a collaborative effort to *O*-propargyl derivatives of glucose and galactose sugars and their reactions with polyfluoro­alkyl azides as a route to novel fluorous surfactants (Ahmed *et al.*, 2020 ▸). Related sugar-derived oligo-propargyl ethers have participated in intra­molecular 1,3-dipolar nitrone addition (Ghorai *et al.*, 2005 ▸) and been used as versatile building blocks in diversity-oriented synthesis of macrocycles (Maurya & Rana, 2017 ▸), while oligo-propargylated sugars and other polyols have been used with oligoazides in a modular approach to neoglycoconjugates (Perez-Baldaras *et al.*, 2009 ▸). Similar vicinal propargyl ethers derived from furfural have found use in conversion to bis­phenols (Hashmi *et al.*, 2007 ▸). Furthermore, dipropargyl malonate and terephthalate esters generate di- and tetra­nuclear clusters with cobalt, molybdenum and ruthenium metal ions (Zhang *et al.*, 2001 ▸). In more tangential, although not exclusive, applications, cyclo­addition reactions of bridged di­acetyl­enic com­pounds have been used to generate a wide range of benzenoid substances, including fluoranthenes and indeno­corannulenes (Wu *et al.*, 2006 ▸), and propargylic enediyne alcohols have shown participation in nucleophilic cyclo­aromatization (Poloukhtine *et al.*, 2010 ▸) akin to the important Bergman cyclization (Bergman, 1973 ▸). Relevant to these topics has been the lengthy and sometimes vexed discourse in the literature over the nature of C—H⋯O hydrogen bonds in crystals (Bernstein, 2013 ▸), where often the donor inter­actions of terminal acetyl­enic groups have been quoted. Matters of contention have been the acid strength and linearity of C—H⋯O hydrogen bonds (Desiraju, 1990 ▸, 1991 ▸), the distinction between weak attractive hydrogen bonds *versus* repulsive van der Waals inter­actions (Steiner & Desiraju, 1998 ▸, 1999 ▸; Schwalbe, 2012 ▸) and the attribution of contacts to electrostatics com­pared with van der Waals inter­actions (Steiner, 2002 ▸; Desiraju, 2002 ▸). Controversy over such matters has subsided (Bernstein, 2013 ▸), with an acceptance that in the solid state there is a continuum of these factors in play, and the best measure of weak donor–acceptor *D*—H⋯*A* contact effectiveness is the *D*⋯*A* distance (D). Such considerations have influenced subsequent applications of weak hydrogen bonds to mol­ecular recognition in organic crystals (Dunitz & Gavezzotti, 2005 ▸), virtual screening in drug design (Desiraju, 2005 ▸; Jones *et al.*, 2012 ▸) and crystal engineering (Desiraju, 2013 ▸, Baillargeon *et al.*, 2014 ▸).

**Figure 1:**
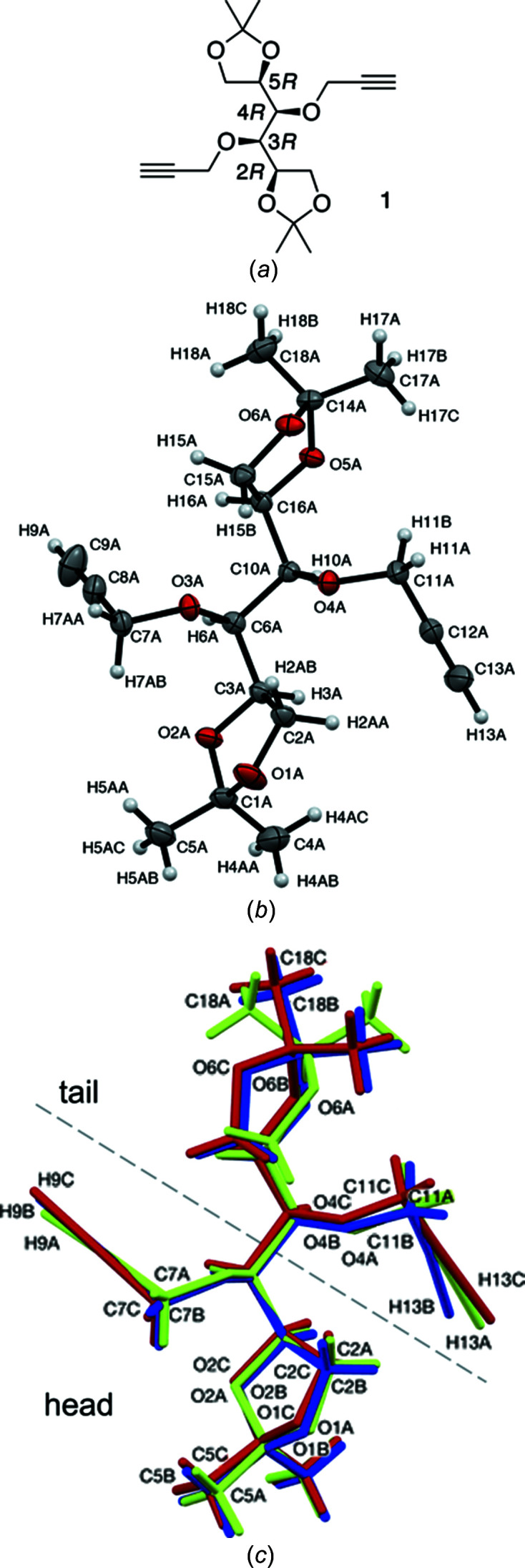
(*a*) The mol­ecular structure of title com­pound **1**, showing the mannitol 2*R*,3*R*,4*R*,5*R* con­figuration. (*b*) The mol­ecular structure of mol­ecule *A*, showing the crystallographic atom-numbering scheme used throughout the remainder of the discussion, with displacement ellipsoids drawn with *Mercury CSD* (Version 3.0; Macrae *et al.*, 2020 ▸) at the 40% probability level and H atoms shown as small spheres of arbitrary radius. (*c*) Overlay of mol­ecules *A* (green), *B* (blue) and *C* (red) from the unit cell of com­pound **1** represented in capped sticks format, showing good overlap in the O1/O2 dioxolanyl portion (head) and significant variation, especially by mol­ecule *A*, in the O5/O6 dioxolanyl portion (tail).

As a homochiral vicinal bis-propargyl ether, substance **1** [Fig. 1 ▸(*a*)] therefore has potential to serve as a precursor for a wide range of intriguing materials whose function would depend largely upon tertiary structure and inter­molecular inter­actions. Its solid-state structure also holds inter­est because of the close proximity of two notionally equivalent terminal acetyl­enic groups in the presence of two ethereal oxygen types (proparg­yloxy and dioxolan­yl) as acceptors. The mol­ecule was reprepared here and examined for the first time by single-crystal X-ray diffraction to ascertain a baseline for these structural features in the solid state.

## Experimental

### Synthesis

The synthesis of 3,4-bis-*O*-propargyl-1,2:5,6-di-*O*-iso­pro­pyl­idene-d-mannitol, **1** {systematic name: (1*R*,2*R*)-1,2-bis­[(*R*)-2,2-dimethyl-1,3-dioxolan-4-yl]-1,2-bis­(prop-2-yn-1-yl­oxy)ethane}, has been reported (Mohammed *et al.*, 2012 ▸) and the X-ray diffraction sample crystallized from EtOAc/*n*-hexane as colourless prisms (m.p. 50–52 °C).

### Refinement

Crystal data, data collection and structure refinement details are summarized in Table 1 ▸. The H atoms were not located in the difference Fourier map. Instead, the H atoms were placed geometrically and constrained according to their environment.

### Analyses of the Cambridge Structural Database (CSD)

#### Searches of the CSD based on *Mercury* Crystal Packing Features (PFF)

A total of 33 individual searches of the Cambridge Structural Database (CSD; Groom *et al.*, 2016 ▸) for Crystal Packing Features (referred to here as PFFs) and illustrated in Fig. S1 (see supporting information) were carried out on 11 unique sets of donor *D* (A1, A2, B, C and D) and acceptor *A* (E, F, G, H, I and J) propargylic contacts that were recognised within the crystal structure of com­pound **1** (Fig. S2). Search criteria specified consideration of ‘Cyclicity’ and were given a ‘Low’ setting tolerance ‘Level of Geometric Similarity’. Where bifurcation was evident, individual PFF searches were performed for each partner pair and then for the two inter­actions together. The output of each search was recorded with a Positive result (a numerical and itemized list of known structures, with structure codes, that fell within the Low Level of Geometric Similarity), and a Negative result (included a corresponding numerical and itemized list of known structures containing the com­ponents of the search query, but where the geometric tolerances were not met). The reference codes of structures regarded as Positive and Negative hits under each PFF search result, and their total numbers and percentages, were com­piled into *Microsoft Excel* spreadsheets. A spreadsheet of the results with matching Positive and Negative structure codes aligned (with the exception of search B1.2) was constructed (Table S2), and the numerical data summarized in graphical form (see Section 3.4.2).

#### Searches based on liberally defined structural motifs using the *ConQuest* search tool

Loosely constrained structural motifs derived from those shown in Fig. S2 were established in the *ConQuest* search tool for propargylic donor inter­actions: CSM_A1, CSM_A2, CSM1_R1, CSM1-R2, CSM1_R3 and CSM1_R4; and acceptor inter­actions: CSM1_R5 and CSM1_R6 (Fig. S3). Relevant distance parameters, D1 (H⋯*A*, Å) and D2 (*D*⋯*A*, Å), were liberally defined as within the sum of the van der Waals radii plus 1.0 Å, and the angular measurements, ANG (*D*—H⋯*A*, °), limited to within 60–180°. Where multiple contacts were recorded for a single com­pound, sometimes within the same category, these were included for com­pleteness. The values for D1, D2 and ANG for all matching contacts found in the CSD were recorded and the results displayed as scatterplots against ‘Identity Number’ in Fig. S3 and discussed more fully in Section 3.4.3.

## Results and discussion

### Mol­ecules in the unit cell

The asymmetric unit com­prises three independent mol­ecules (represented as *A*-green, *B*-blue and *C*-red), each with the mannitol 2*R*,3*R*,4*R*,5*R* con­figuration [Fig. 1 ▸(*b*), randomly selected mol­ecule *A*] and differing only in the con­formation of the mol­ecule. This establishes asymmetry in each mol­ecule. Hence, in future, and for ease of reference, the terms ‘head’ and ‘tail’ sections will be used for each mol­ecule, based on lower and higher crystallographic element numbers, respectively [Fig. 1 ▸(*c*)].

#### Conformations of mol­ecules *A*–*C*

Based on torsion angles (Table 2 ▸), there are marginal differences in the con­formation about the central mannitol core (Entries 1–3), with mol­ecule *A* the most notable. Marginal differences are also observed in torsions associated with the orientation of the dioxolanyl groups relative to the core (Table 2 ▸, Entries 4–15), but most notably in mol­ecule *A* and to the largest extent in the tail portion of the mol­ecules. In contrast, puckering of the dioxolanyl group, as reflected in the C1/14—O3/10—C3/16—C6/10 torsion angles (Table 2 ▸, Entries 16–21), was most varied in the head section, and to the most significant extent (moderately) in mol­ecule *C*; con­figurations in the tails of mol­ecules *A*–*C* were highly consistent. The proparg­yloxy substituents potentially have three sources of con­formational freedom. Marginal differences are observed in the torsions associated with their attachment to the mannitol core in both head and tail sections (Table 2 ▸, Entries 22–27), but most noticeably in mol­ecule *A*. From this point, the orientation of the propargyl groups in com­parison with the mannitol core are relatively conserved in the heads and tails of all three mol­ecules (Table 2 ▸, Entries 28–33). However, significant differences are then observed in the orientations of the terminal acetyl­enic groups in the head and tail sections relative to the mannitol fragment (Table 2 ▸, Entries 34–39), with the most extreme difference appearing through atom O3 in the head of mol­ecule *A* and atom O4 in the tail of mol­ecule *B*.

### Strand and sheet assemblies

#### Recognition of the like mol­ecular strand construct

**Figure 2:**
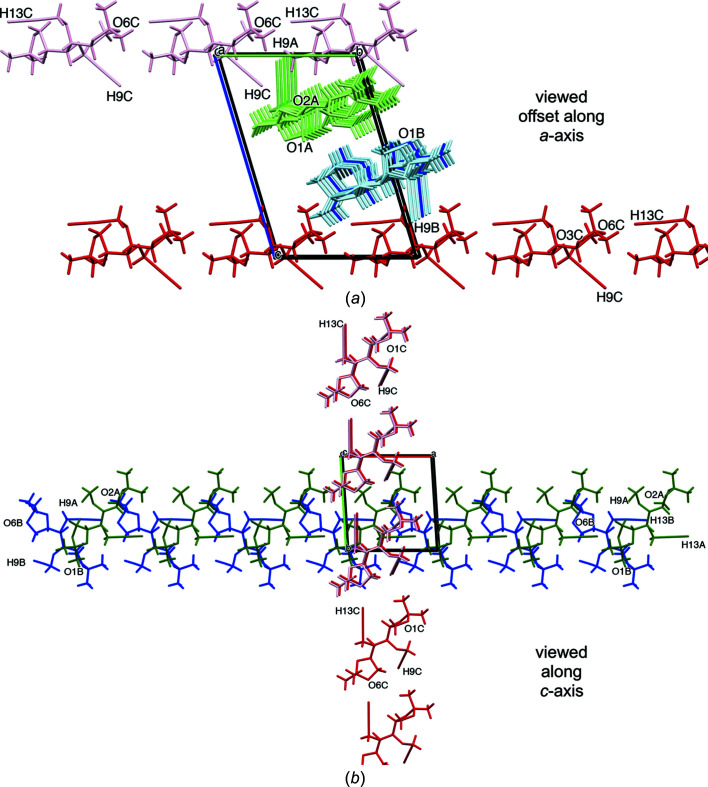
Individual crystal packing of mol­ecules *A*, *B* and *C* in com­pound **1**. (*a*) View slightly off-set from the *a* axis, showing the near linear (θ = 167.8°) alignment of tail-to-tail C13*C*—H13*C*⋯O6*C* intra­strand contacts and inter­digitation inter­actions through inter­strand donor propargylic H9*A* (green) and H9*B* (blue) atoms with acceptor dioxolanyl atoms O6*C* (red) and ether atoms O3*C* (red), respectively; measurement of very weak engagement between inter­strand donor proparygyl H9*C* (red) and acceptor propargylic ether atoms O2*A* (green) is not shown here, but discussed in Section 3.2.3. (*b*) View along the *c* axis showing the parallel, unidirectional and tail-to-tail arrangements of strands of outstretched mol­ecules *A* (green) and *B* (blue), and the orthogonal tail-to-tail arrangement of strands of mol­ecules *C* (red), all with acetyl­enic donor C13—H13⋯O6 dioxolanyl acceptor contacts; the positions of atoms C5 and H9 are also shown for reference purposes. Generic atom labels without symmetry codes have been used.

More detailed analysis reveals that, in the crystal, the three independent species (*A*-green, *B*-blue and *C*-red) align in strands, each with matching identical mol­ecules *A*, *B* and *C*. Mol­ecules in each strand engage through unique tail-to-tail inter­actions. The differences in each case appear to arise because of subtle differences in the con­formations of each mol­ecule. Such individual strands all occur through C13—H13⋯O6 contacts and are represented and viewed along the *a* axis in Fig. 2 ▸(*a*) and along the *c* axis in Fig. 2 ▸(*b*). Thus, the contacts for mol­ecules *A* (green) and *B* (blue) occur in a unidirectional sense along the *b* axis, with identical symmetry codes, namely (*x* + 1, *y*, *z*), while strands of mol­ecules *C* (red) are oriented perpendicular, along the *a* axis [consider Figs. 2 ▸(*a*) and 2 ▸(*b*)], through a different symmetry code, (*x*, *y* − 1, *z*). As is also evident, particularly in Fig. 2 ▸(*b*) and the positions of atoms C5 and C9, the mol­ecules in strands *A* and *B* are flipped relative to each other by approximately 180° about the length of the two strands. This feature permits near coincidence of the C13—H13⋯O6 alignments, with unidirectionality of the overall strands, while accommodating the steric demands of the remaining portions of the mol­ecules.

#### Homogeneous mol­ecular strands and sheets

**Figure 3:**
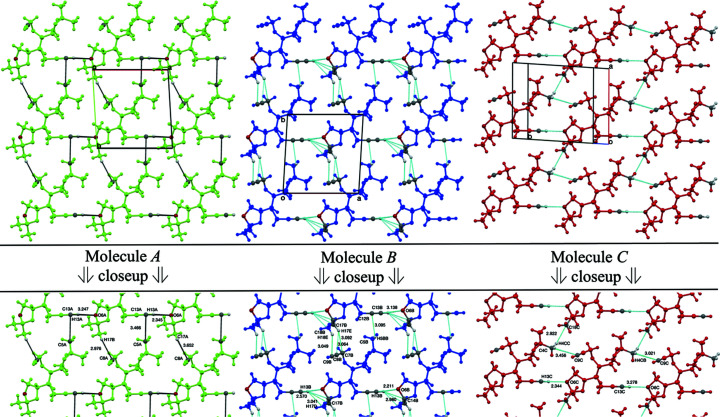
Inter- and intra­strand contacts between like mol­ecules of strands *A*, *B* and *C*, as seen from directly above the mean planes of the *A*, *B* and *C* sheets, with unit cells indicated, as well as closeup views with labels and distance measurements included.

*3.2.2.1. Intra­strand contacts between like mol­ecules.* Significant advances have been made towards estimating the H-atom positions of small organic mol­ecules from X-ray crystallographic data (Jayatilaka & Dittrich, 2008 ▸; Capelli *et al.*, 2014 ▸; Woińska *et al.*, 2016 ▸). These have achieved results that are within the accuracy of neutron diffraction. However, a very limited number of examples have been described. As a consequence, contact data for short intra- and inter­strand inter­actions between like mol­ecules of each type have been summarized in Table 3 ▸ using parameters that have become accepted more widely as good indicators of strength and efficiency in the field for weak hydrogen bonds (Desiraju, 2005 ▸). The values include the accurately measured *D*⋯*A* distance (D, Å), the estimated *D*—H bond length using the previously utilized predicted position of the H atom for each type of weak acid species [CH (acetyl­enic) = 0.95 Å, CH (methine at *sp* ^3^ carbon) = 1.00 Å, CH_2_ (methyl­ene) = 0.99 Å and CH_3_ (meth­yl) = 0.98 Å], without any additional normalization, and the resulting H⋯*A* distance (Å). Common to each of the strands are the aforementioned intra­strand tail-to-tail C13—H13⋯O6 contacts (Table 3 ▸; Entries 1, 4 and 12). All three achieve short *D*⋯*A* distances with near-linear *D*—H⋯*A* contact angles (*A* 158.3, *B* 164.7 and *C* 167.8°). Observed values are consistent with acetyl­enic groups with p*K*_*a*_ (Me_2_SO) ∼ 24.9 (Pedireddi & Desiraju, 1992 ▸). These intra­strand associations stand alone for mol­ecule *A*, but are reinforced within the strands from mol­ecule *B* by additional more distant C13—H13*B*⋯C14*B*, C13—H13*B*⋯C17*B* and C17*B*—H17*D*⋯H13*B* contacts (Table 3 ▸, Entries 5–7), and within those from mol­ecule *C* by an equally distant head-to-head C4*C*—H4*CB*⋯C9*C* contact (Table 3 ▸, Entry 13) (see also Fig. 3 ▸). Those involving mol­ecule *B* are exceptionally weak, as assessed by *D*⋯*A* distance measurements. They stem from additional engagement by the C13*B*—H13*B* group as a donor in a trifurcated contact with quaternary atom C14*B* and its neighbouring bonded methyl C17*B* and H17*D* atoms. The additional intra­strand contact involving mol­ecules *C* is remarkable because it occurs between highly remote groups that ordinarily are weak donors and acceptors.

*3.2.2.2. Inter­strand contacts between like mol­ecules.* Inter­strand contacts are also observed between like mol­ecular strands (Table 3 ▸ and Fig. 3 ▸). These vary between parallel strands of mol­ecules *A*, *B* and *C* with inter­strand spacings, as measured by C13⋯C13′ distances of 10.300 (5), 10.300 (5) and 9.473 (4) Å, respectively, to create planar sheets of singular mol­ecular type (Fig. 3 ▸). Donors in each case com­prise normally weak dioxolanyl methyl groups, in which those between strands of *A* and *B* resemble each other, and those between strands *C* engage differently. For example, those from mol­ecule *A* include a noticeably moderate C5*A*⋯C13*A* contact [*D*⋯*A* = 3.466 (4) Å] (Table 3 ▸, Entry 2) orthogonal to the aforementioned C13*A*—H13*A*⋯O6*A* inter­action, while those from mol­ecule *B* include a similar but specific C5*B*—H5*BB*⋯C12*B* contact [*D*⋯*A* = 3.612 (3) Å] (Table 3 ▸, Entry 8), neither of which are observed between the strands of mol­ecule *C*. Similarly, mol­ecule *A* participates in a weaker C17*A*—H17*B*⋯C8*A* contact with a propargylic acceptor (Table 3 ▸, Entry 3), while the equivalent donor group in mol­ecule *B*, namely, C17*B*—H17*E*, engages in a bifurcated hydrogen-donor arrangement with the propargylic C7*B*—C8*B* bond (Table 3 ▸, Entries 9 and 10), neither of which are evident in the strands of mol­ecule *C*. Mol­ecule *B* is also involved in a separate propargylic H18*E*⋯C9*B* inter­action (Table 3 ▸, Entry 11), which is absent in the strands of *A*, but is observed indirectly in those of mol­ecule *C* (Fig. 3 ▸). Thus, mol­ecule *C* shows a weak inter­strand donor C4*C*—H4*CC*⋯C18*C* contact [*D*⋯*A* = 3.867 (4) Å], as well as the medium-strength intra­strand C4*C*—H4*CB*⋯C9*C* contact [*D*⋯*A* = 3.456 (4) Å] (Table 3 ▸, Entries 14 and 13, respectively).

A consequence of these analyses is that, despite the orthogonal alignment of strands of mol­ecules *C* relative to those of mol­ecules *A* and *B*, the two-dimensional array of short contacts between like mol­ecules in com­pound **1** is found to result in the formation of homogenous sheets of each mol­ecular type.

#### Cross-strand/cross-sheet inter­actions

Separate mol­ecular inter­actions occur between, rather than within, the sheets of mol­ecules *A*–*C*. The noncon­forming orientation of the *C* strand com­pared with the *A* and *B* strands, in particular, led us to examine even more closely this aspect of the supra­molecular structure. In addition, the inter­molecular inter­actions involving the propargylic C9—H9 acetyl­ene donor functional groups in the head moieties were of inter­est. Contact data for these cross-strand/cross-sheet contacts (contacts between donors and acceptors from different mol­ecular types *A*–*C*), almost all of which are ostensibly stronger C—H⋯O contacts, are summarized in Table 4 ▸. Contact angle (θ, °), as well as distance measurements, are given for added depth of analyses. Values for intra­strand acetyl­enic C13—H13⋯O6 contacts are included for com­parison (Table 4 ▸, Entries 1–3). As with the earlier analyses, it is acknowledged that the resulting data are derived from a single-crystal X-ray crystallographic study and not a com­prehensive crystallographic database search. However, the observations and conclusions drawn from them are based on com­parisons from well debated past literature.

**Figure 4:**
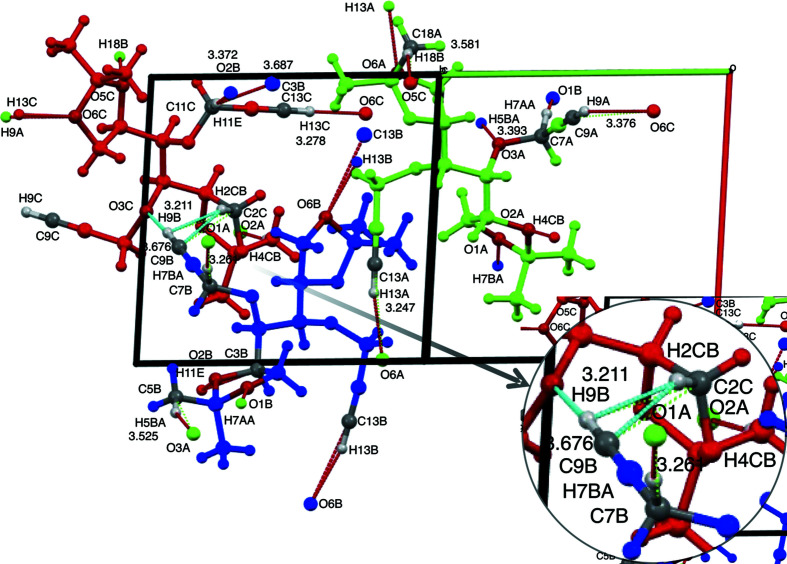
The unit cell of compound **1** viewed down the crystallographic *b* axis after rotating by 135° about the vertical axis, showing molecules *A* (green), *B* (blue) and *C* (red) with selected atom labels of close contacts as defined automatically within the limits of van der Waals radius −0.05 to 0.30 Å, within the crystal lattice, and an enlarged inset with details of the linear and orthogonal contacts involving C9*B*—H9*B* and C2*C*—H2*CB* with additional enforced distance measurements to C2*C* indicated. Note that the C9*C*—H9*C*⋯O2*A* contact was not detected under the conditions set for close contacts.

Cross-strand acetyl­enic C9—H9 donor inter­actions occur from mol­ecules *A*, *B* and *C* (Table 4 ▸, Entries 4–6). Superficially, those from mol­ecules *A* (green) and *B* (blue) engage in unique finger-like intrusions, that are almost perpendicular to the axis of the parent strand, into separate strands of mol­ecules *C* (red) [Fig. 2 ▸(*b*)], yet each one of the three contacts is different in detail. Readers will find Fig. 4 ▸ helpful in providing a pictorial view of the short contacts within the unit cell, as expressed in Table 4 ▸.

**Figure 5:**
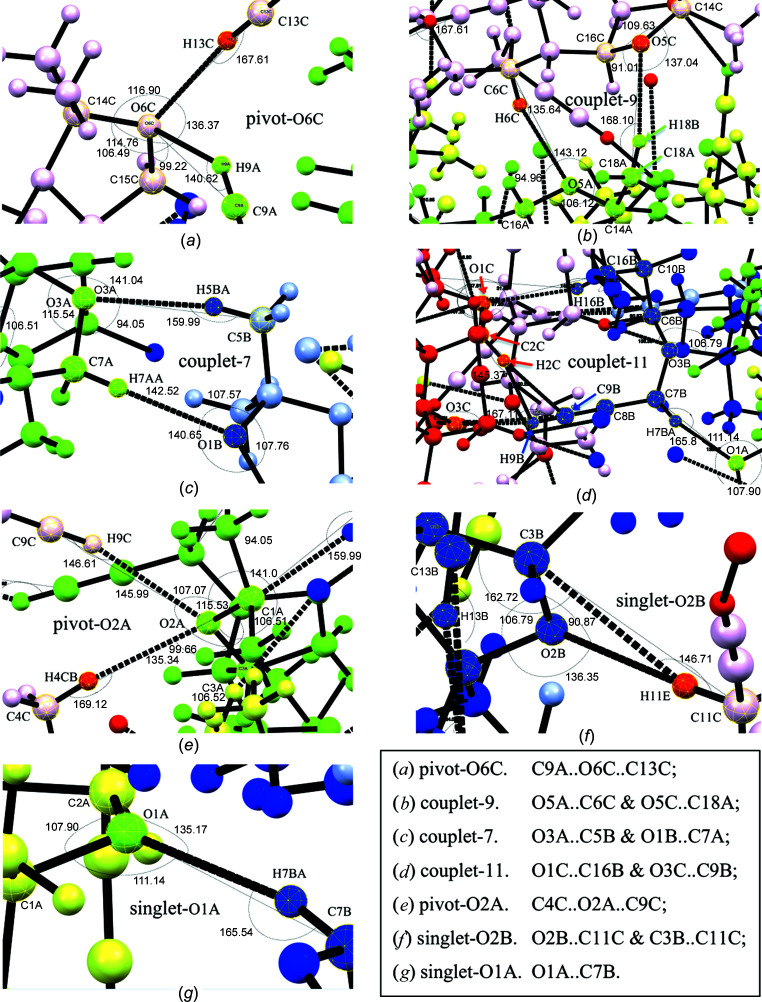
Major types of oxygen-acceptor-centred short inter­strand contacts in the X-ray crystal structure of com­pound **1**, with key atomic labels and bond angles, as recorded by *Mercury* (Version 2020.3.0; Macrae *et al.*, 2020 ▸), for mol­ecules *A* (green), *B* (blue) and *C* (red).

*3.2.3.1. Cross-strand inter­actions from the standpoint of donor elements.* Inter­strand inter­actions are grouped in Table 4 ▸ according to notional donor acid strength, as defined by calculated *D*—H bond lengths, which are based on donor-atom electronegativity. This classification does not correlate directly with the understood mark of contact strength, namely, *D*⋯*A* distance, even when contact angle (θ) is considered. The inter­actions in Table 4 ▸ are therefore discussed in this order, but within the context of three inter­action types: *singlet*, *pivot* and *couplet* (see Fig. 5 ▸).

Mol­ecules *A* (green) provide a modest cross-strand C9*A*—H9*A* acetyl­enic donor inter­action with the tail dioxolanyl O6*C* atom in addition to the strong intra­strand, C13*C*—H13*C* donor contact with the same acceptor (Table 4 ▸, Entries 3 and 4). Because the acceptor O6*C* serves as a fulcrum in bringing together adjacent *A* and *C* mol­ecules, the contact is called here a ‘*pivot*’ inter­action [Fig. 5 ▸(*a*)]. The short-to-medium C9*A*—H9*A*⋯O6*C* contact distance [D = 3.376 (4) Å] is consistent with a slightly weaker acid than the acetyl­enic C13—H13 groups (Pedireddi & Desiraju, 1992 ▸). Its contact angle (140.8°) (Table 4 ▸, Entry 4) is far from the near linear alignment of its partner (167.8°; Table 4 ▸, Entry 3) but within the range of other contacts from weak acids where inter­actions have been attributed to electrostatics (Desiraju, 1990 ▸; Pedireddi & Desiraju, 1992 ▸). However, in this pivot case, where both donors are of the same chemical type, the different *D*⋯*A* distances is most likely a reflection of the different contact angles, with the linear contact being more dominant.

In a separate pivot inter­action [Fig. 5 ▸(*e*)], mol­ecules *C* (red) demonstrate a somewhat longer [D = 3.541 (2) Å] acetyl­enic donor contact (C9*C*—H9*C*⋯O2*A*; Table 4 ▸, Entry 6), identified only by a directed measurement (therefore not visible in Fig. 4 ▸) with the head dioxolanyl group of an *A* mol­ecule. The inter­action is associated with another nonlinear contact angle (146.6°), but in this case made with a near linear contact (169.1°) with its pivot donor partner C4*C*—H4*C*B (Table 4 ▸, Entry 17). The donor in this portion of the pivot is derived from a dioxolanyl methyl group, which would normally be a much less acidic proton source than a terminal acetyl­ene group (com­pare the estimated *D*—H bond lengths of 0.98 *versus* 0.95 Å). The observed *D*⋯*A* distances for the two inter­actions are indicative of the anti­cipated donor strengths, but closer in magnitude than one might expect. One explanation for their similarity is the acute angle of the first, which would diminish the donor effectiveness from p*K*_*a*_, and linearity of the latter, which would enhance effectiveness from p*K*_*a*_ for the methyl H atoms that are already the most acidic of those attached to *sp* ^3^ C atoms in com­pound **1**.

Contrasting these dioxolanyl contacts, mol­ecules *B* (blue) participate in extremely short donor acetyl­enic C9*B*—H9*B* inter­actions [D = 3.121 (3) Å] with the head O3*C* propargylic ether O atom of mol­ecules *C* (red) (see Fig. 4 ▸ and enlargement), with an accordingly near-linear contact angle (167.1°) (Table 4 ▸, Entry 5). The acetyl­enic C9*B*—H9*B* bond is also engaged in a secondary near-orthogonal contact with the nearby C2*C*—H2*C* bond (Fig. 4 ▸). This geometry is supported by the H9*B*—C9*B*⋯C2*C*—H2*CB* torsion angle (φ = 83.8°; not recorded in Table 2 ▸) and small contact angles associated when the *D*—H group is considered C9*B*—H9*B* (107.4 and 112.1°; Table 4 ▸, Entries 7–8). However, the widely differing contact distance measurements (Table 4 ▸, Entries 7–10) for H⋯*A* (D = 2.35–2.78 Å) and *D*⋯*A* [D = 2.78–3.676 (3) Å] reflect a highly distorted orthogonal cluster. The closer contact between participants H2*CB*⋯C9*B* (2.78 Å) than H9*B*⋯C2*C* (3.21 Å) and larger contact angle values with C2*C*—H2*CB* as the *D*—H group (145.5 and 150.3°) (Table 4 ▸, Entries 9–10) are most consistent with C2*C*—H2*CB*⋯C9*B* being the main contact. This secondary inter­action is suggestive of a bifurcated H2*CB* (Desiraju, 1991 ▸), which probably contributes to the extreme shortness of the C9*B*⋯O3*C* distance.

These contacts, together, contribute to another type of co-operative set of contacts, called here a ‘*couplet*’, that include in this example the non-acetyl­enic dioxolanyl methine C16*B*—H16*B*⋯O1*C* contact [Table 4 ▸, Entry 16; Fig. 5 ▸(*d*)]. This is one of three couplet inter­actions [Figs. 5 ▸(*b*)–(*d*)] observed in the crystals of com­pound **1** that, by definition, bring together two inter­strand partners through two independent *D*⋯*A* contacts. In this example, the bifurcated H-atom donor from C2*C*—H2*CB* and its contact with the acetyl­enic C9*B* as an acceptor contribute to a ‘symmetrical’ (donor and acceptor in each mol­ecular contributor) 11-membered ring of couplet atoms. Alternatively, the bifurcated H atom can be considered as contributing to a wider 15-membered ‘unsymmetrical’ couplet involving the C9*B*—H9*B*⋯O3*C* contact; in this situation, the two donor com­ponents participate from *B* mol­ecules while the acceptors are located in *C* mol­ecules in an unsymmetrical alliance. Couplets of either type can limit con­formational flexibility and distort normal contact angles or, through their attractive nature, force contacts closer together. In the case of the methine C16*B*—H16*B*⋯O1*C* inter­action, the *D*⋯*A* contact distance [D = 3.584 (3) Å] (Table 4 ▸, Entry 16) is somewhat longer than for the only other methine contact, C6*C*—H6*C*⋯O5*A* (Entry 15), for which the contact angle is smaller. However, it is nearly identical to those of the pivot partners around O2*A* (Table 4 ▸, Entries 6 and 17), wherein contact angles are unequal. It is also very similar to those of methyl donor contacts C5*B*—H5*B*⋯O3*A* and C18*A*—H18*B*⋯O5*C* (Entries 18 and 19) with more linear contact angles. The medium-to-large *D*⋯*A* distance in the case of C16*B*—H16*B*⋯O1*C* is possibly the result of it being part of a reasonably large couplet of atoms, and its contact angle the result of con­finement of the donor C16*B*—H16*B* bond as part of the tail dioxolanyl ring system in mol­ecule *B*.

Three non-acetyl­enic donor types of C—H⋯O close contacts are recognisable within the distance range initially defined, and all are cross-strand (see Fig. 3 ▸). Those recorded as Entries 11–14 in Table 4 ▸ are considered in the first category because they involve similarly weakly acidic *D*—H participants (note the longer estimated *D*—H distances for Entries 9–14 com­pared with those for the acetyl­enic donor examples in Entries 1–8). They proceed from the methyl­ene groups at atoms C7 and C11 in the head and tail proparg­yloxy substituents, respectively, but their inter­actions are in turn each different.

Mol­ecules *A* and *B* make reciprocal head-group methyl­ene contacts with the dioxolanyl O1 acceptor from the partner mol­ecule. Notably, the donor from mol­ecule *B* (Entry 12) makes a ‘*singlet*’ contact with O1*A* [Fig. 5 ▸(*g*)], that is, a contact without the involvement of any other partner. The singlet C7*B*—H7*BA*⋯O1*B* contact is closer [D = 3.261 (2) Å] and more linear (165.8°) than the C7*A*—H7*AA*⋯O1*B* contact from mol­ecule *A* [Entry 11; D = 3.393 (2) Å, θ = 142.5°], which is part of another symmetrical seven-membered couplet [Fig. 5 ▸(*c*)] with the donor methyl C5*B*—H5*BA*⋯O3*A* inter­action [Entry 18, D = 3.525 (3) Å, θ = 160.0°]. The smaller ring size of this tight couplet appears to impart a more acute angle to the C7*A*—H7*AA*⋯O1*B* contact and thereby must reduce the *D*⋯*A* distance. In contrast, only C11*C*—H11*E* participates as donor from the equivalent methyl­ene group of mol­ecule *C*, but the same donor makes contact with two quite different acceptors, namely, dioxolanyl O2*B* and C3*B*, with very different *D*⋯*A* distances. However, these acceptors reside at adjacent positions in the same mol­ecule *B* (Table 3 ▸, Entries 13 and 14), and largely for this reason the contact is defined here as a ‘*singlet*’ inter­action [Fig. 5 ▸(*f*)]. As expected, because the acceptor O2*B* has two frontier orbitals, each with lone pairs of electrons, contact with O2*B* is much shorter and more linear [Entry 13, D = 3.372 (3) Å, θ = 176.9°] with respect to the predicted C11*C*—H11*E* bond than it is with the adjacent methine C3*B* acceptor [Entry 14, D = 3.687 (4) Å, θ = 146.7°], with no free bonding electron pairs. The fact that atom H11*E* is bifurcated probably accounts for the slightly longer C11*C*⋯O2*B* contact com­pared with the C7*B*⋯O1*A* contact, but with a distance that is tempered by the near perfect alignment of the former.

The second type of non-acetyl­enic donor close contacts exhibit methine donor contributions from C6*C*—H6*C* and C16*B*—H16*B*, respectively (Table 4 ▸, Entries 15 and 16). Contacts occur with inter­strand dioxolanyl oxygen partners, but with only moderate *D*⋯*A* distances (D) and contact bond angles (θ). Both C atoms are derived from the mannitol skeleton, the first bearing a proparg­yloxy substituent and the other an oxygen substituent that is part of the tail dioxolanyl group. It is possibly significant that none of mol­ecules *A*–*C* exhibit close contacts involving donor C—H bonds from either of the symmetry-equivalent atoms C10 and C3, respectively, of these positions. However, it is worth recalling that C3*B* does participate in a contact with donor C11*C*—H11*E* (Table 4 ▸, Entry 14), but only as a weak acceptor, and then with limited efficiency. Donor C16*B*—H16*B* was also mentioned earlier as a close contact with O1*C* within the first couplet com­plex [Fig. 5 ▸(*d*)], with C9*B*—H9*B*⋯O3*C*. The remaining methine donor contact, C6*C*—H6*C*⋯O5*A* (Table 4 ▸, Entry 15), participates in an unsymmetrical nine-membered couplet with a tail–tail methyl donor contact, C18*B*—H18*B*⋯O5*C* [Table 4 ▸, Entry 19; Fig. 5 ▸(*b*)]. Perhaps because of its slightly different methine donor character, the C6*C*—H6*C*⋯O5*A* contact has a measurably shorter *D*⋯*A* distance [D = 3.457 (2) Å] than the C16*B*—H16*B*⋯O1*C* contact [D = 3.584 (3) Å] (Table 4 ▸, Entries 15 and 16). Equally, the difference in *D*⋯*A* contact distances might arise from the smaller contact angle for C6*C*—H6*C*⋯O5*A* brought about by constraints of its smaller ring couplet than those of the larger one involving C16*B*—H16*B*⋯O1*C*. Finally, the three remaining non-acetyl­enic donor contacts emanate from a C—H bond in one of the slightly more acidic, axial or equatorial geminal methyl groups attached to the dioxolanyl groups (Table 4 ▸, Entries 17–19). The three donors inter­act either head–head or tail–tail with a dioxolanyl O-atom acceptor and have com­parable *D*⋯*A* distances (D) with near-linear contact angles (θ). All three contacts have been discussed above within the context of other inter­actions (Table 4 ▸, Entries 6, 15, and 16).

**Figure 6:**
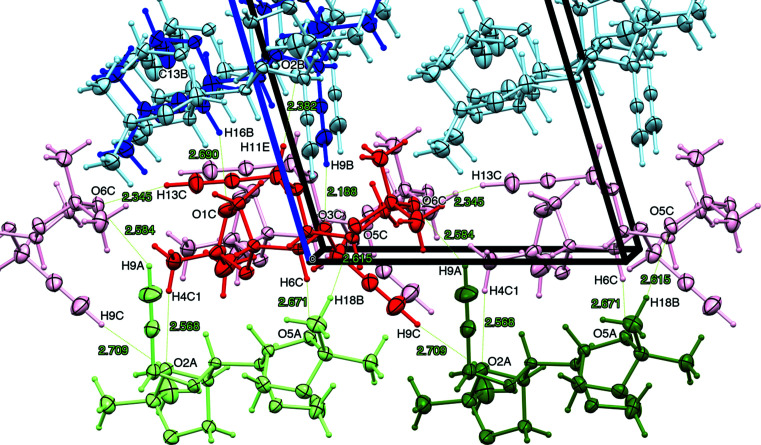
Close up of com­pound **1**, viewed along the *a* axis, with a slight offset showing differing inter­digitation of C9—H9⋯O inter­actions between layers of independent mol­ecules *A* (green), *B* (blue) and *C* (red), represented with non-H atoms as ellipsoids for clarity; key C—H donor partners are shown in darker colours.

Between them, this com­plex array of contacts affords stability to the observed alternating layered sheets of *A* (green)–*C* (red)–*B* (blue) mol­ecules (Fig. 6 ▸). The arrangement leaves no inter­digitation of propargyl groups between layers of mol­ecules of type *A* (green) and *B* (blue). However, there are alternative *A*⋯*B* reciprocal C—H⋯O contacts (Table 4 ▸, Entries 11–12 and 18), of which the head-to-head propargylic C7*B*—H7*B*⋯O1*A* contact and nonpropargylic C5*B*—H5*BA*⋯O3*A* contact are most important.

With this improved understanding of contacts from the perspective of C—H donors, a brief study was made of the geometry about the most important cross-strand contact acceptors in com­pound **1**, the relevant dioxolanyl and proparg­yloxy ether O atoms.

*3.2.3.2. Cross-strand inter­actions from the standpoint of O-atom acceptors.* Data derived from measurements of individual bond and contact angles associated with covalently bound O atoms and their close inter­molecular donor C—H contacts are summarized in Table 5 ▸. This process was initiated on the questionable premise that acceptor inter­actions would take place through O-atom lone pairs of electrons (Taylor & Kennard, 1982 ▸, 1984 ▸; Steiner & Desiraju, 1998 ▸) and with the intention of providing better insight into the geometry at the acceptor sites.

The method used acknowledges that the O atoms in mol­ecule **1** are all ethers and should have an electron-pair geometry that is approximately tetra­hedral with coordinate angles of 109.5°. Accordingly, the sum of the triplet of bond and contact angles surrounding each relevant acceptor O atom has been calculated and the geometry arbitrarily assessed as ‘pyramidal’ (trigonal pyramidal) or ‘planar’, depending on whether the angle sum is less or more, respectively, than 344°, midway between the ideal for tetra­hedral (328.5°) and planar (360°). In this study, close donor–acceptor (*D*⋯*A*) contacts are limited to those shorter than the sum of the van der Waals radii minus 0.01 Å. Under these conditions, atoms O2*C*, O3*B*, O4*A*–O4*C* and O5*B* showed no close contacts. In Table 5 ▸, the values of the *D*⋯*A* distance (D, Å) and contact angle (θ, °) of each observed C—H⋯O short contact are repeated from Table 4 ▸ and the contact types from Fig. 5 ▸ are added, all for reference purposes and as an aid to inter­pretation.

Surprisingly, only six of the 14 contacts with O atoms can be classified as pyramidal in their geometry. While all these are associated with dioxolanyl O atoms, not all the dioxolanyl O-atom contacts can be classified in this way. As partly discussed in the context of donors, in two of the six cases, dioxolanyl atoms O6*C* (tail) and O2*A* (head) each makes pivot contacts with two C—H donors (Table 4 ▸, Entries 3/4 and 6/17, respectively). This gives the inter­actions a degree of com­plexity, but with some hope of understanding differences in the geometry at the fulcrum, which is their O atom. In the first case [Table 5 ▸, Entries 3 and 4; Fig. 5 ▸(*a*)], two of the angular com­ponents used to evaluate the pyramidal or planar geometry about O6*C* are nearly identical, but the C15*C*—O6*A*⋯*D* angle differs markedly, *i.e.* 135.4 (1)° when *D* = C13*C* and 99.2 (1)° when *D* = C9*A*. The rendition of these contacts in Fig. 5 ▸(*a*) gives insight into the com­petition by donors C13*C*—H13*C* and C9*A*—H9*A* for access to O6*C*. It also gives an understanding as to how the closer more linear contact resulting from the former might have arisen through an overall planar geometry with O6*C*, with a splaying of the C15*C*—O6*A*⋯C13*C* angle, and a slightly weaker more apical contact at O6*C* through an acute angular inter­action (probably electrostatic) by C9*A*—H9*A*. In the second pivot example [Table 5 ▸, Entries 9 and 10; Fig. 5 ▸(*e*)], a similar inter­play appears to be involved, but the less acidic donor partner, C4*C*—H4*CB*, exerts a closer than expected near-linear contact with acceptor O2*A* at an angle sum [341.5 (2)°] that is close to being defined as that for a planar contact. As a result, the C1*A*—O2*A*⋯C4*C* and C3*A*—O2*A*⋯C9*C* com­ponent angles are increased and the C9*C*—H9*C*⋯O2*A* contact weakened concomitantly with a decrease in the observed contact angle to 146.6°.

Analyses of observed geometries around each of the O-atom acceptors in the three sets of couplet inter­actions reported in Table 5 ▸ (Entries 5–8 and 13–14) reveal that the observations are consistent with similar com­promises in individual contributing angular com­ponents, contact distances and contact angles, but with additional consideration of the nature and size of the couplet. For example, in the symmetrical nine-membered couplet involving O5*A* and O5*C* [Fig. 5 ▸(*b*)], the unequal length of the carbon bridge between the donor and acceptor in mol­ecule *A* (four atoms) and mol­ecule *C* (five atoms) causes a severe enlargement of the inter­nal C14*A*—O5*A*⋯C6*C* angle [143.1 (1)°] at the expense of the C16*A*—O5*A*⋯C6*C* angle [95.0 (1)°]. At the same time, the connectivity of the two *D*⋯*A* systems imposes the reverse distortion of the corresponding individual angles around O5*C*, with a com­pression of the inter­nal C16*C*—O5*C*⋯C18*A* angle [91.0 (1)°] at the expense of the C14*C*—O5*C*⋯C18*A* angle [137.0 (1)°]. The resulting shorter *D*⋯*A* contact for the former [D = 3.457 (2) Å] takes place through an acute *D*—H⋯*A* angle (135.6°) and planar though angularly distorted inter­action with O5*A*. This result occurs despite the more linear (168.1°) contact of the normally more acidic methyl donor C18*A*—H18*B* with its couplet partner O5*C*. Analysis of the seven-membered couplet [Table 5 ▸, Entries 7 and 13; Fig. 5 ▸(*c*)] provides a similarly satisfying explanation for angular distortions around the O1*B* and O3*A* acceptors and indicates a more convincing dominance of one contact, the C7*A*—H7*AA*⋯O1*B* inter­action, over the other.

The situation in the 11-membered couplet [Table 5 ▸, Entries 8 and 14; Fig. 5 ▸(*d*)] is more com­plex because of the participation of the orthogonal donor arrangement of the bifurcated H2*C* atom, but it is clear that equally explicable distortions of contributing angles around key acceptor com­ponents O1*C*, C9*B* and O3*C* do take place as a result of the couplet arrangement of the two partner com­ponents. Equally, tolerated distortions around the O2*B* and O1*A* acceptor O atoms in the two singlet cases [Table 5 ▸, Entries 11 and 12; Figs. 5 ▸(*f*) and 5(*g*)] are explicable for the simple reasons of neighbouring-atom participation and crystallographic dislocation of participants.

### Crystal packing

A review of the crystallographic data to this point highlights a number of noteworthy features about com­pound **1**. The three independent mol­ecules *A*–*C* that make up the unit cell differ subtly in con­formation, but significantly at two of their ether sites. They each assemble into unique linear strands of like mol­ecules, primarily through C13—H13⋯O6 contacts, but supported by intra­molecular and intra­strand inter­actions. Furthermore, the assemblies are unidirectional, with the strands of *A* and *B* aligned in close proximity, head-to-tail, along the crystallographic *a* axis and those of *C* aligned orthogonal along the *b* axis. Additional inter­strand inter­actions between like mol­ecules establish a two-dimensional sheet array of like parallel strands. However, a network of donor–acceptor contacts occur between strands/sheets of unlike mol­ecular type.

#### Mol­ecular strand and sheet planes

**Figure 7:**
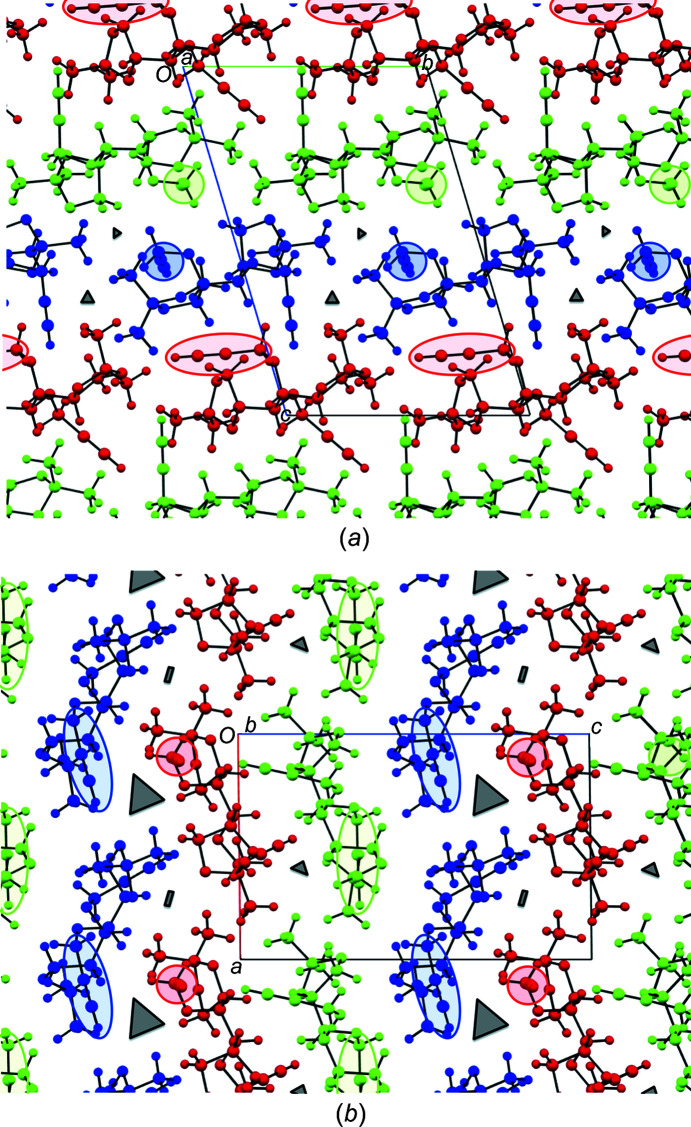
Crystal packing diagrams for com­pound **1**, showing the distinct layering of independent mol­ecules *A* (green), *B* (blue) and *C* (red), as viewed in (*a*) along the *a* axis and in (*b*) along the *b* axis; the propargylic group in the ‘tail’ moiety of each mol­ecule is circled in its respective colour to highlight the orthogonal directionality of the *C* (red) com­pared with the *A* (green) and *B* (blue) groups. Void spaces observed in the space-filling models are illustrated in cartoon form by dark-grey shapes with relative sizes drawn approximately to scale.

Initial examination of the crystal packing reveals a repeat layering of the three mol­ecular types in the order *A* (green)–*B* (blue)–*C* (red), when viewed along the *a* and *b* axes (Fig. 7 ▸). Analysis of the mean planes of each mol­ecule across three separate strands con­firms their parallel arrangement, which is most convincing in Fig. 7 ▸(*a*). Such layering is consistent with the establishment of sheets (Section 3.2.2.2).

**Figure 8:**
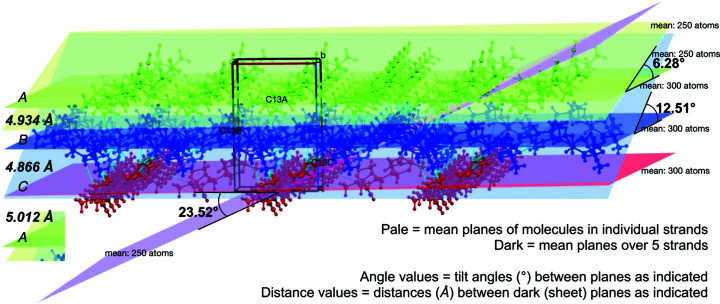
Mean planes of mol­ecules *A* (green), *B* (blue) and *C* (red), as viewed slightly offset along the crystallographic *b* axis and recorded (*a*) in pale shades for individual mol­ecular strands showing nonparallel but like planes for *A* and *B*, which are orthogonal to the angular plane of *C* strands, and (*b*) in dark shades for mean planes over five strands of each type, showing parallel but unequally spaced layers, which are repeated in the same order throughout.

Further analysis of the mean planes of the *A*, *B* and *C* mol­ecules in their respective strands across a span of five mol­ecules in each strand reveals tilts of 6.28, 12.51 and 23.52°, respectively, from the mean planes of the sheets of each mol­ecular type, which are themselves separated unequally by *A*⋯*B* = 4.934 Å, *B*⋯*C* = 4.866 Å and *C*⋯*A* = 5.012 Å (Fig. 8 ▸).

In Fig. 7 ▸, the C13—H13-bearing propargyl group in each mol­ecule is highlighted by encircling the group. Collectively the orientations of the encircled groups reinforce their orthogonality in the *A* and *B* strands relative to those in the *C* strands. The intrusion of the equivalent C9—H9-bearing propargyl groups from mol­ecules *A* and *B* into the strands of mol­ecule *C* and reciprocal angular intrusion of the group from mol­ecule *C* only into strands of mol­ecule *A* is noticeable in Fig. 7 ▸(*a*), and accounts for the minor differences in inter-sheet spacings. This leaves very little inter­action between mol­ecules *A* and *B*, as is evident in Fig. 7 ▸(*b*) and as was discussed in Section 3.2.3.1. Minor void spaces are visible, especially in Fig. 7 ▸(*b*), but these are too small for any mol­ecular inclusions.

### CSD searches based on *Mercury* Crystal Packing Features and *ConQuest* search motifs involving donor and acceptor acetyl­enic contacts observed in com­pound 1.

#### Background

In the preceding diffraction studies of com­pound **1**, intra- and inter­molecular contacts were observed in which acetyl­enic com­ponents of the two propargyl groups participated in various situations as C—H donors and as C—H acceptors. Two principal searches of the Cambridge Structural Database (CSD; Groom *et al.*, 2016 ▸) were carried out in order to ascertain the prevalence of these inter­actions and their scope in crystal engineering. These com­prised firstly a *Mercury*-based study using highly constrained contact motifs derived from its Crystal Packing Feature (PFF) (Fig. S1) using measurements taken directly from com­pound **1** (Fig. S2). The second study utilized the *ConQuest* search tool and more loosely defined motifs (CSM) (Fig. S3) that, while artificial in their construct, were again based on general inter­pretations of the observed contacts (Fig. S2). The outcomes of these searches are discussed separately.

#### Analysis of *Mercury* Crystal Packing Feature (PFF) search results

**Figure 9:**
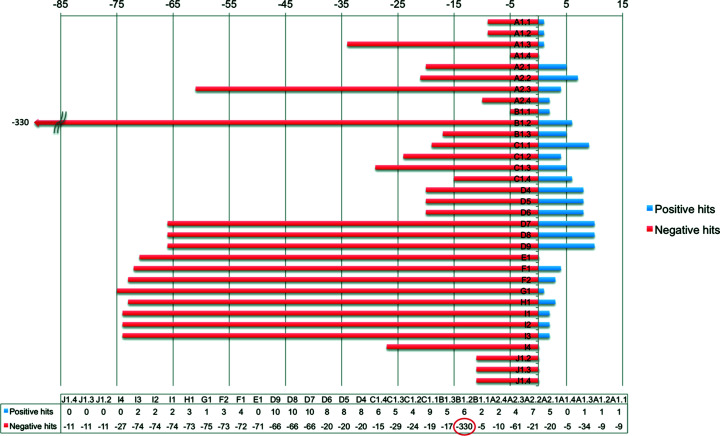
A bar graph and table of the number of Positive (blue) and Negative (red) PFF results from each of the PFF searches.

As a general observation from the results summarized in Fig. 9 ▸ and Table S2, the propargylic group gave more positive matches when it participated as a donor through its terminal acetyl­enic proton than when the group served as a proton acceptor (Fig. 9 ▸). An analysis of findings from each contact type is described in detail in the supporting information, and summarized in the following sections.

*3.4.2.1. Propargyl group as donor.* There were considerable differences when the propargyl group served as a donor. Searches C and D were the more populous in terms of positive and negative results, while search B was extremely variable, especially with respect to negative results. Searches were dependent upon *D*⋯*A* distances, with the observed shorter distances of stand-alone strand-forming contacts in group A being less common than equivalent inter­strand contact distances or distances involving shared contacts with adjacent acceptor atoms. This dependence showed strong variation with the nature of the acceptor atom and with the number and extent of prescription, including Cyclicity, in the atoms/groups associated with the acceptor atom.

*3.4.2.2. Propargyl group as acceptor.* Despite many fewer positive results from motifs E–J than from motifs A–D, the number of negative results from searches remained in excess of 70 in cases E–I3, and there was less scope than in the A–D cases for varying attached groups to either *D* or *A* atoms. Cases G1.1 and H1.1 provided situations with equivalent *D* and *A* types where minor differences in numbers of positive results (1 *versus* 3, respectively, perfectly counterbalanced by the differences in negative results, 75 *versus* 73) were observed. It was not possible to determine if these resulted intrinsically from very slightly higher *D*⋯*A* contact distances, or ultimately by the significantly different *D*—H⋯*A* bond angles brought about by inter­molecular intra­strand (G1.1) com­pared with inter­strand (H1.1) contacts (Fig. 3 ▸ and Table 4 ▸). This situation was helped marginally when the E1.1, F1.1 and F2.1 inter­actions were considered as a whole (Fig S1 and Table 3 ▸). The E1.1 and F2.1 features showed a similarity not evident in F1.1; the bond to the distal C atom to which their donor methyl groups are attached is nearly unidirectional to the axis of the methyl C atom to acetyl­enic C atom trajectory, while in F1.1 it is at an acute angle (Fig. S1). Despite this observation, individual analyses showed that the more accurately measureable *D*⋯*A* distances increased in the order F2.1 ≤ E1.1 << F1.1, which was not the same as the order of the H⋯*A* distances (F2.1 ≤ F1.1 << E1.1) or the *D*—H⋯*A* angles (E1.1 < F2.1 << F1.1). On the other hand, for E1.1 there were no positive results but 71 negative results, and for F1.1 and F2.1, both recorded 76 results, with four and three, respectively, recorded as positive.

It was concluded from the lack of direct correlation between any of these trends, including intra­stand and inter­strand inter­actions, and the observed number of positive results, that the E1.1, F1.1 and F2.1 features are equally common to those in G, H and I, in the solid state. Again, the *D*⋯*A* distances encountered in the crystal structure of com­pound **1** must impose tight limitations that are not commonly met in structures within the CSD.

*3.4.2.3. Analysis of structure codes for negative search results.* Analysis of the breakdown of structure codes from each search (Table S2) showed relatively good coherence in the structure codes in the negative results for categories A1, A2 and C–I, but not for B1.1–B1.3. This outcome appears to mark a change from cyclic to acyclic O-atom acceptors. A similar lack of coherence was observed, *albeit* to a less dramatic extent because of fewer overall search results, for PFF J1.2–J1.4. Here it was noted that the donor H atom was part of a cyclic methyl­ene group rather than from an exocyclic methyl group. Such factors were therefore important in inter­preting the negative search results.

*3.4.2.4. Analysis of structure codes for positive search results.* As for positive results, there was a degree of coherence between structure codes within each of the search PFFs A1.1–A1.4, A2.1–A2.4, B1.1–B1.3 and C1.1–C1.4. The differences that were observed were readily attributable to variations in the attachments to the common acceptor atom in each set. In contrast, there was no overall coherence in the codes in the positive results between the first three categories, *i.e.* A1, A2 and B1 (Table S2). Initial thoughts of donor type or *D*⋯*A* distance as the cause were ruled out. Instead, a much more subtle feature appeared to be at play, namely, a different type of O-atom acceptor (Fig. S2), the influence of which was not as evident in the negative results. The similarities in positive result structure codes between A2, C and D results could then be explained by H⋯C inter­actions in PFF C and D that were strongly influenced by the presence of the corresponding dioxolanyl-derived O—CH_2_ attachment to the formal quaternary C-atom acceptors.

#### Analysis of loosely constrained *ConQuest* structural motif (CSM) search results

Despite the predominance of positive propargylic donor over acceptor inter­actions in strand assemblies in the crystal structure of com­pound **1**, the absolute sum of positive and negative donor inter­actions in each category from the study in Section 3.4.2 remained remarkably small. This prompted a more general search of the CSD for less constrained structural motifs (Fig. S3) that encom­passed the main features of those already examined but focused on H⋯*A* (D1) and *D*⋯*A* (D2) contact distances, and *D*—H⋯*A* (ANG) angles.

*3.4.3.1. CSD Index Numbers *versus* contact distances and angles.* Simple scatterplots of the individual D1, D2 and ANG values *versus* the CSD Index Numbers, with their respective search structure motifs (Fig. S3), revealed different cluster patterns in the distances, and to some degree contact angles, of each category, but no direct correlations, particularly between cases of multiple independent contacts within the one structure.

**Figure 10:**
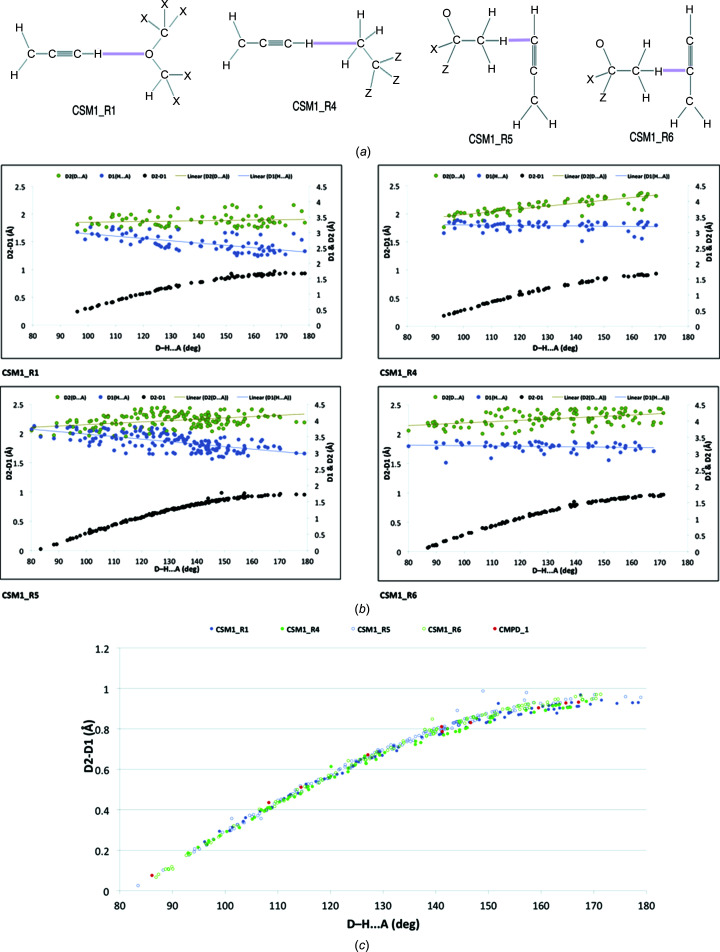
(*a*) *ConQuest* search motifs (CSMs) used to define searches of propargylic donor and acceptor inter­actions using loosely constrained distances (D1 and D2) and *D*—H⋯*A* angles (ANG). (*b*) Scatterplots of D1 (blue), D2 (green) and D2–D1 (black)(Å) values *versus* ANG (°) values for inter­actions in mol­ecules satisfying criteria for *ConQuest* search motifs CSM1-R1, CSM1-R4, CSM1-R5 and CSM1-R6, including lines of best fit for the D1 and D2 results. (*c*) Overlay of the four scatterplots of D2–D1 values *versus* contact angle (ANG) from Fig. 10 ▸(*b*) (with changed marker shapes and colours), as well as related data points (in red) for relevant contacts in com­pound **1**.

*3.4.3.2. Contact distances and distance differences *versus* contact angles.* Far more useful patterns emerged when scatterplots were constructed of D1 and D2 distances *versus D*—H⋯*A* (ANG) contact angles for the most populous donor acetyl­enic contacts to O (CSM1_R1) and C (CSM1_R4) acceptor atoms on the one hand, and acceptor acetyl­enic contacts at terminal C atoms (CSM1_R5) and C atoms adjacent to the terminal C atoms (CSM1_R6) by *sp* ^3^ C—H donors on the other [Fig. 10 ▸(*a*)].

All searches gave noticeable differences between D1 and D2 that became larger with increasing contact angles [upper portions of each plot in Fig. 10 ▸(*b*)]. Initial observations were codified by additionally recording scatterplots of D2–D1 values against contact angles for each contact motif [lower portions of each plot in Fig. 10 ▸(*b*)]. These showed a nonlinear progression of larger D2–D1 values with increasing contact angle. However, calculated trend lines for each set of the D1 and D2 distance curves unmasked stark differences for each search category in the contributions of D1 and D2. For example, at the two extremes, the CSM1_R1 inter­actions involved a relatively constant D2 (*D*⋯*A*) distance and decreasing D1 (H⋯*A*) distances, while those of the CSM1_R4 and CSM1_R6 inter­actions showed the opposite, with relatively constant D1 and decreasing D2 distances. In CSM1_R5, the D2 (*D*⋯*A*) distances increased marginally, while the D1 (H⋯*A*) distances decreased noticeably, with increasing *D*—H⋯*A* angle, indicating that both parameters contributed. Neither absolute magnitudes of D1 and D2 in each search category, which fell in the order CSM1_R1 < CSM1_R4 < CSM1_R5 ≃ CSM1_R6, nor reported contact angles, which fell within four different ranges, could account for these observations. Instead, it was concluded that the type of acceptor atom (O *versus* C and terminal *versus* nonterminal acetyl­enic C) was probably responsible.

Despite these anomalies, when the scatterplots of the numerical difference (D2–D1) in contact distances *versus* contact angle in each category were plotted together, the correlation curves were virtually superposable [Fig. 10 ▸(*c*)]. Modelling studies revealed that very minor variations in the correlation curves were attributable to the different fixed C—H donor bond lengths (0.95–1.00 Å) embedded in each data set. Of the seven data points that could be regarded as outliers from these acknowledged trends, six were attributable to features in the CSD structures for just one com­pound, WUJWAC {(*R*)-1-[(4*S*,5*R*)-5-(hy­droxy­meth­yl)-2,2-dimethyl-1,3-dioxolan-4-yl]but-3-yn-1-ol} (Heinrich *et al.*, 2020 ▸) (1 × CSM1_R1, 1 × CSM1_R4, 3 × CSM1_R5 and 1 × CSM1_R6), and one attributable to one other com­pound, EHAKAZ [*N*-((1*R*,2*S*)-2-hy­droxy-1-{(4*S*,4′*R*,5*S*)-2,2,2′,2′-tetra­methyl-[4,4′-bi(1,3-dioxolan)]-5-yl}pent-4-yn-1-yl)acetamide] (Liu *et al.*, 2002 ▸) (CSM1_R5) (Fig. S4). Capture of the outliers was due to the liberal contact criteria inherent in the searches. The origins of their outlier properties could not be ascertained, although both mol­ecules possess chirality and contain acetonide (dioxolan­yl) and alcohol groups with multiple opportunities for additional com­peting inter- and intra­molecular contacts. In particular, the hydroxyl groups of diol WUJWAC participitate in numerous strong hydrogen-bond inter­actions in the crystal, which probably drive the very com­plex array of weaker close contacts.

Relevant to the present study, an overlay of the corresponding measured data for representatives of contacts from the three crystallographic mol­ecules *A*–*C* from com­pound **1** showed perfect superposition, with marker points [Fig. 10 ▸(*c*)] in red that were dispersed within the normal scatter across the full angular range of the data from the CDS searches.

To our knowledge, the type of com­parisons just described have not been reported previously. They support the notion that acetyl­enic groups, particularly those originating in propargylic substituents, can participate in a wide range of weak but highly influential donor and acceptor inter­actions that are important in establishing crystal packing. These contacts can be mediated over a large range of contact angles, even within crystals of the one com­pound. It is valuable to recognize the high consistency of the correlations participated in by the terminal acetyl­enic com­ponent of the propargyl group, both as donor and as acceptor. As a corollary, rare departures from this norm can be an indication of additional powerful influences that might be present.

## Summary and conclusion

A com­prehensive single-crystal X-ray crystallographic analy­sis of 3,4-bis-*O*-propargyl-1,2:5,6-di-*O*-iso­propyl­idene-d-man­ni­tol, **1**, has revealed the presence of three independent mol­ecules *A*–*C* in the unit cell, each differing in con­formation. The mol­ecules form a close-packed layered structure aligned in the *a* and *b* axes, with each layer com­prising a well-ordered homogeneous array of like mol­ecules. Tail-to-tail acetyl­enic C13—H13 donor and dioxolanyl O6 acceptor contacts are associated with strand-like substructures in each layer. Multiple, much weaker, inter­strand contacts are associated with the packing of parallel strands in each sheet. Strands derived from mol­ecules *A* and *B* align co-operatively with minimal contact, along the crystallographic *a* axis, while those from *C* align orthogonally along the *b* axis. A thorough systematic analysis of intra- and inter­molecular inter­actions, including an examination of the geometric parameters associated with the observed close contacts, and consideration of the crystallographic planes, allows identification of the key inter­actions and provides strong support for the current under­standing of weak hydrogen bonds and their description as a continuum of van der Waals contacts and electrostatic inter­actions. The evidence supports the notion that contact strength is best assessed in a *D*—H⋯*A* system from the *D*⋯*A* distance (D, Å), with a considerable flexibility in the *D*—H⋯*A* contact angle and the geometry about the acceptor, at least when *A* is oxygen or carbon.

Two secondary studies of the Cambridge Structural Database (CSD) using *Mercury* Crystal Packing Features (PFF) and *ConQuest* structural motifs, based on features identified in the crystals of com­pound **1** involving the propargyl group, add further insight into the value of **1** as a model for the study of weak inter­actions in the solid state. They give mathematical credence to the close correlation that exists in these *D*—H⋯*A* systems between the difference in distance between *D*⋯*A* and H⋯*A*, and the *D*—H⋯*A* angle, but point to different contributions that the *D*⋯*A* and H⋯*A* parameters can have in this correlation, depending upon the particular structural motif involved.

Overall, the studies described here provide new insight into factors involved in weak acetyl­enic H⋯*A* inter­actions and might well prove useful in guiding the design of chemoselective applications of such functional groups, especially where these are propagated in or close to the solid state.

## Supplementary Material

Crystal structure: contains datablock(s) I, global. DOI: 10.1107/S205322962200897X/oc3016sup1.cif

Structure factors: contains datablock(s) I. DOI: 10.1107/S205322962200897X/oc3016Isup2.hkl

Supporting information file. DOI: 10.1107/S205322962200897X/oc3016Isup3.mol

Supplementary Information with experimental methods, crystallographic analysis criteria and graphical results. DOI: 10.1107/S205322962200897X/oc3016sup4.pdf

CCDC reference: 2055328

## Figures and Tables

**Table 1:** Experimental details

Crystal data	
Chemical formula	C_18_H_26_O_6_
*M*_r_	338.39
Crystal system, space group	Triclinic, *P*1
Temperature (K)	150
*a*, *b*, *c* (Å)	9.4726 (4), 10.3000 (5), 15.3583 (7)
α, β, γ (°)	73.378 (2), 88.382 (2), 86.400 (2)
*V* (Å^3^)	1432.94 (11)
*Z*	3
Radiation type	Mo *K*α
μ (mm^−1^)	0.09
Crystal size (mm)	0.24 × 0.21 × 0.19

Data collection	
Diffractometer	Bruker APEXII CCD
Absorption correction	Multi-scan (*SADABS*; Bruker, 2016 ▸)
*T*_min_, *T*_max_	0.679, 0.746
No. of measured, independent and observed [*I* > 2σ(*I*)] reflections	25389, 8343, 8134
*R*_int_	0.028
(sin θ/λ)_max_ (Å^−1^)	0.595

Refinement	
*R*[*F* ^2^ > 2σ(*F* ^2^)], *wR*(*F* ^2^), *S*	0.026, 0.065, 1.02
No. of reflections	8343
No. of parameters	661
No. of restraints	3
H-atom treatment	H-atom parameters constrained
Δρ_max_, Δρ_min_ (e Å^−3^)	0.21, −0.15
Absolute structure	Flack *x* determined using 3228 quotients [(*I* ^+^) − (*I* ^−^)]/[(*I* ^+^) + (*I* ^−^)] (Parsons *et al.*, 2013 ▸)
Absolute structure parameter	0.04 (17)

Computer programs: *APEX3* (Bruker, 2016 ▸), *SAINT* (Bruker, 2016 ▸), *SHELXT* (Sheldrick, 2015*a* ▸), *SHELXL* (Sheldrick, 2015*b* ▸) and *OLEX2* (Dolomanov *et al.*, 2009 ▸).

**Table 2:** Selected torsion data for the alignment of head and tail dioxolanyl and proparg­yloxy groups, with each other and independently relative to the central mannitol chain of each of the crystallographically independent mol­ecules *A*–*C* in the unit cell of com­pound **1**, showing individual torsion angles (φ), mean values within each head and tail group, and observed deviations from the means*^*a*^*

Entry	Position	Torsions	Angle, φ (°)	*A*–*C* Mean, φ (°) (±deviation)
1	central	C3*A*—C6*A*—C10*A*—C16*A*	−176.7 (2)	180.0 (2.3)*^*b*^*
2	central	C3*B*—C6*B*—C10*B*—C16*B*	175.2 (2)	180.0 (−4.8)*^*b*^*
3	central	C3*C*—C6*C*—C10*C*—C16*C*	175.5 (2)	180.0 (−4.8)*^*b*^*
				
		C—C3/16—C6/10—C	Dioxolan­yl(C)—C relative to core(C)	
4	head	C2*A*—C3*A*—C6*A*—C10*A*	76.6 (2)	78.7 (−2.1)
5	head	C2*B*—C3*B*—C6*B*—C10*B*	80.6 (2)	78.7 (1.9)
6	head	C2*C*—C3*C*—C6*C*—C10*C*	78.9 (2)	78.7 (0.2)
7	tail	C6*A*—C10*A*—C16*A*—C15*A*	85.0 (2)	77.4 (7.6)
8	tail	C6*B*—C10*B*—C16*B*—C15*B*	73.8 (2)	77.4 (−3.6)
9	tail	C6*C*—C10*C*—C16*C*—C15*C*	73.3 (2)	77.4 (−4.1)
				
		O—C3/16—C6/10—C	Dioxolan­yl(O)—C relative to core(C)	
10	head	O2*A*—C3*A*—C6*A*—C10*A*	−167.1 (2)	−165.0 (−2.1)
11	head	O2*B*—C3*B*—C6*B*—C10*B*	−163.4 (2)	−165.0 (1.6)
12	head	O2*C*—C3*C*—C6*C*—C10*C*	−164.4 (2)	−165.0 (0.6)
13	tail	C6*A*—C10*A*—C16*A*—O5*A*	−159.5 (2)	−166.8 (7.3)
14	tail	C6*B*—C10*B*—C16*B*—O5*B*	−170.2 (2)	−166.8 (−3.4)
15	tail	C6*C*—C10*C*—C16*C*—O5*C*	−170.6 (2)	−166.8 (−3.8)
				
		C—O2/5—C3/16—C	Dioxolan­yl(C)—O relative to core(C)	
16	head	C1*A*—O2*A*—C3*A*—C6*A*	−152.2 (2)	−148.2 (−4.0)
17	head	C1*B*—O2*B*—C3*B*—C6*B*	−149.9 (2)	−148.2 (−1.7)
18	head	C1*C*—O2*C*—C3*C*—C6*C*	−142.4 (2)	−148.2 (5.3)
19	tail	C14*A*—O5*A*—C16*A*—C10*A*	−137.4 (2)	−137.9 (0.5)
20	tail	C14*B*—O5*B*—C16*B*—C10*B*	−139.2 (2)	−137.9 (−1.3)
21	tail	C14*C*—O5*C*—C16*C*—C10*C*	−137.0 (2)	−137.9 (0.9)
				
		O—C6/10—C10/6—C	Proparg­yl(O) relative to core(C)	
22	head	O3*A*—C6*A*—C10*A*—C16*A*	−52.4 (2)	−58.2 (5.8)
23	head	O3*B*—C6*B*—C10*B*—C16*B*	−61.4 (2)	−58.2 (−3.2)
24	head	O3*C*—C6*C*—C10*C*—C16*C*	−60.8 (2)	−58.2 (−2.6)
25	tail	C3*A*—C6*A*—C10*A*—O4*A*	−54.4 (2)	−58.7 (4.3)
26	tail	C3*B*—C6*B*—C10*B*—O4*B*	−61.0 (2)	−58.7 (−2.3)
27	tail	C3*C*—C6*C*—C10*C*—O4*C*	−60.7 (2)	−58.7 (−2.0)
				
		C—O3/4—C6/10—C	Proparg­yl(C)—O relative to core(C)	
28	head	C7*A*—O3*A*—C6*A*—C10*A*	148.0 (2)	148.1 (−0.1)
29	head	C7*B*—O3*B*—C6*B*—C10*B*	149.0 (2)	148.1 (0.9)
30	head	C7*C*—O3*C*—C6*C*—C10*C*	147.4 (2)	148.1 (−0.7)
31	tail	C11*A*—O4*A*—C10*A*—C6*A*	142.6 (2)	140.3 (2.3)
32	tail	C11*B*—O4*B*—C10*B*—C6*B*	138.2 (2)	140.3 (−2.1)
33	tail	C11*C*—O4*C*—C10*C*—C6*C*	140.1 (2)	140.3 (−0.2)
				
		C—O3/4—C7/11—C	Proparg­yl(C)—C relative to core(C)	
34	head	C6*A*—O3*A*—C7*A*—C8*A*	−58.5 (2)	−68.9 (10.4)
35	head	C6*B*—O3*B*—C7*B*—C8*B*	−72.9 (2)	−68.9 (−4.0)
36	head	C6*C*—O3*C*—C7*C*—C8*C*	−75.3 (2)	−68.9 (−6.4)
37	tail	C10*A*—O4*A*—C11*A*—C12*A*	−86.2 (2)	−79.4 (−6.8)
38	tail	C10*B*—O4*B*—C11*B*—C12*B*	−63.2 (2)	−79.4 (15.2)
39	tail	C10*C*—O4*C*—C11*C*—C12*C*	−88.7 (2)	−79.4 (−9.3)

Notes: (*a*) the colours highlight the torsions of most difference within each triplet: red 2–7°, green 7–12° and blue >12°. (*b*) Deviation from the ideal angle of 180°.

**Table 3:** Short intra­strand and inter­strand contacts between like mol­ecules from each of strands *A*–*C*

*Strand*/Entry	Intra/inter strand	*D*⋯*A* positions	*D*—H⋯*A*	*D*—H	H⋯*A*	*D*⋯*A*	Contact angle, θ
*A*							
1	intra	tail–tail	C13*A*—H13*A*⋯O6*A* ^i^	0.95	2.34	3.247 (3)	158.3
2	inter	head–tail	C5*A*—H5*A*A⋯C13*A* ^ii^	0.98	3.39	3.466 (4)	86.1
3	inter	tail–head	C17*A*—H17*B*⋯C8*A* ^ii^	0.98	2.98	3.652 (3)	127.1
							
*B*							
4	intra	tail–tail	C13*B*—H13*B*⋯O6*B* ^i^	0.95	2.21	3.138 (2)	164.7
5	intra	tail–tail	C13*B*—H13*B*⋯C14*B* ^i^	0.95	2.98	3.884 (3)	159.5
6	intra	tail–tail	C13*B*—H13*B*⋯C17*B* ^i^	0.95	3.04	3.827 (4)	141.2
7	intra	tail–tail	C17*B*—H17*D*⋯H13*B* ^i^	0.98	2.57	3.04	109.5
8	inter	head–tail	C5*B*—H5*BB*⋯C12*B* ^ii^	0.98	3.10	3.612 (3)	114.4
9	inter	tail–head	C17*B*—H17*E*⋯C7*B* ^ii^	0.98	3.09	3.919 (4)	143.0
10	inter	tail–head	C17*B*—H17*E*⋯C8*B* ^ii^	0.98	3.06	3.812 (4)	134.1
11	inter	tail–head	C18*B*—H18*E*⋯C9*B* ^ii^	0.98	3.05	3.861 (5)	141.1
							
*C*							
12	intra	tail–tail	C13*C*—H13*C*⋯O6*C* ^ii^	0.95	2.34	3.278 (3)	167.8
13	intra	head–head	C4*C*—H4*CB*⋯C9*C* ^ii^	0.98	3.02	3.456 (4)	108.3
14	inter	head–tail	C4*C*—H4*CC*⋯C18*C* ^iii^	0.98	2.92	3.867 (4)	162.5

Symmetry codes: (i) *x* − 1, *y*, *z*; (ii) *x*, *y* − 1, *z*; (iii) *x* − 1, *y* + 1, *z*.

**Table 4:** Short-contact donor (*D*) acetyl­enic (Entries 1–8) and non-acetyl­enic (Entries 9–18) H⋯acceptor (*A*) inter­actions, initially defined automatically within the limits of van der Waals radius −0.05 to 0.30 Å, and measured in Angstroms (Å), for mol­ecules *A*–*C* in the crystal lattice of com­pound **1**, as well as *D*—H⋯*A* contact angles (θ, °), where *A* = oxygen (O) in most cases, and relevant carbon (C) and hydrogen (H) close contacts in other cases, with relevant head and tail denominations for participating groups, useful for indicating the nature of their alignment

Entry	*D*⋯*A* positions	*D*—H⋯*A*	*D*—H	H⋯*A*	*D*⋯*A*	Contact angle, θ
1	tail–tail*	C13*A*—H13*A*⋯O6*A* ^i^	0.95	2.34	3.247 (3)	158.3
2	tail–tail*	C13*B*—H13*B*⋯O6*B* ^i^	0.95	2.21	3.138 (2)	164.7
3	tail–tail*	C13*C*—H13*C*⋯O6*C* ^ii^	0.95	2.34	3.278 (3)	167.8
4	head–tail	C9*A*—H9*A*⋯O6*C* ^iii^	0.95	2.58	3.376 (4)	140.8
5	head–head	C9*B*—H9*B*⋯O3*C*	0.95	2.19	3.122 (3)	167.1
6	head–head^*a*^	[C9*C*—H9*C*⋯O2*A* ^iv^	0.95	2.71	3.541 (3)	146.6]
7	head–head	C9*B*—H9*B*⋯C2*C*	0.95	3.21*^*b*^*	3.676 (3)	112.1
8	head–head	C9*B*—H9*B*⋯H2*C*B	0.95	2.35	2.78	107.4
9	head–head*^*b*^*	C2*C*—H2*CB*⋯C9*B*	0.99	2.78	3.676 (3)	145.4
10	head–head*^*b*^*	C2*C*—H2*CB*⋯H9*B*	0.99	2.35	3.21*^*c*^*	150.3
11	head–head	C7*A*—H7*AA*⋯O1*B* ^v^	0.99	2.55	3.393 (2)	142.5
12	head–head	C7*B*—H7*BA*⋯O1*A* ^ii^	0.99	2.29	3.261 (2)	165.8
13	tail–head	C11*C*—H11*E*⋯O2*B* ^vi^	0.99	2.38	3.372 (3)	176.9
14	tail–head	C11*C*—H11*E*⋯C3*B* ^vi^	0.99	2.82	3.687 (4)	146.7
15	head–tail	C6*C*—H6*C*⋯O5*A* ^vii^	1.00	2.67	3.457 (2)	135.6
16	tail–head	C16*B*—H16*B*⋯O1*C*	1.00	2.69	3.584 (3)	149.1
17	head–head	C4*C*—H4*CB*⋯O2*A* ^vii^	0.98	2.57	3.535 (3)	169.1
18	head–head	C5*B*—H5*BA*⋯O3*A* ^v^	0.98	2.59	3.525 (3)	160.0
19	tail–tail	C18*A*—H18*B*⋯O5*C* ^v^	0.98	2.62	3.581 (3)	168.1

Symmetry codes: (i) *x* − 1, *y*, *z*; (ii) *x*, *y* − 1, *z*; (iii) *x*, *y* − 1, *z* − 1; (iv) *x*, *y* + 1, *z* + 1; (v) *x* − 1, *y* − 1, *z*; (vi) *x* + 1, *y*, *z*; (vii) *x*, *y*, *z* − 1. Notes: (*a*) Not visible in Fig. 4 ▸. (*b*) Non-acetyl­enic donor (*D*) and acetyl­enic acceptor (*A*) (see Fig. 6 ▸). (*c*) The C2*C*⋯H9*B* contact distance falls outside the constraints set for others and the value was obtained by targeted measurement. (*) Denotes an intra­strand inter­action.

**Table 5:** Analysis of the geometry of close inter­molecular contacts, as defined as shorter than the sum of the van der Waals radii minus 0.01 Å, through measurement of triplet com­ponent angles at each of the key O-atom acceptor atoms*^*a*^* in mol­ecules *A*–*C* of com­pound **1**, the arithmetic sum of the individual angles and resulting assignment of con­figuration, and duplicate records of relevant *D*⋯*A* distances (Å) and C—H⋯O contact angles, θ (°), with the contact type

Triplet	*D*⋯*A*	Component angles	Angle (°)	Angle sum (°)	Contact type
Entry	positions			Con­figuration^*b*^	C—H⋯O
				*D*⋯*A* (Å)	Angle (°)
1		C14*A*—O6*A*—C15*A*	106.41 (15)	328.9 (2)	
		C14*A*—O6*A*⋯C13*A*	110.9 (1)	pyramidal	singlet-O6*A*
	tail–tail	C15*A*—O6*A*⋯C13*A*	111.6 (1)	3.247 (3)	158.3
					
2		C14*B*—O6*B*—C15*B*	106.73 (14)	341.8 (2)	
		C14*B*—O6*B*⋯C13*B*	111.0 (1)	pyramidal	singlet-O6*B*
	tail–tail	C15*B*—O6*B*⋯C13*B*	124.1 (1)	3.138 (2)	164.7
					
3		C14*C*—O6*C*—C15*C*	106.49 (14)	358.8 (2)	
		C14*C*—O6*C*⋯C13*C*	116.9 (1)	planar	pivot-O6*C*
	tail–tail	C15*C*—O6*C*⋯C13*C*	135.4 (1)	3.278 (3)	167.8
					
4		C14*C*—O6*C*—C15*C*	106.49 (14)	320.5 (13)	
		C14*C*—O6*C*⋯C9*A*	114.8 (1)	pyramidal	pivot-O6*C*
	head–tail*	C15*C*—O6*C*⋯C9*A*	99.2 (1)	3.376 (4)	140.8
					
5		C14*A*—O5*A*—C16*A*	108.14 (1)	346.2 (1)	
		C14*A*—O5*A*⋯C6*C*	143.1 (1)	planar	couplet-9
	head–tail*	C16*A*—O5*A*⋯C6*C*	95.0 (1)	3.457 (2)	135.6
					
6		C14*C*—O5*C*—C16*C*	109.83 (15)	337.8 (2)	
		C14*C*—O5*C*⋯C18*A*	137.0 (1)	pyramidal	couplet-9
	tail–tail*	C16*C*—O5*C*⋯C18*A*	91.0 (1)	3.581 (3)	168.1
					
7		C6*A*—O3*A*—C7*A*	115.53 (14)	350.5 (1)	
		C6*A*—O3*A*⋯C5*B*	141.0 (1)	planar	couplet-7
	head–head*	C7*A*—O3*A*⋯C5*B*	94.0 (1)	3.525 (3)	160.0
					
8		C6*C*—O3*C*—C7*C*	114.70 (16)	352.3 (2)	
		C6*C*—O3*C*⋯C9*B*	131.0 (1)	planar	couplet-11
	head–head*	C7*C*—O3*C*⋯C9*B*	106.6 (1)	3.122 (3)	167.1
					
9		C1*A*—O2*A*—C3*A*	106.52 (15)	359.6 (2)	
		C1*A*—O2*A*⋯C9*C*	107.1 (1)	planar	pivot-O2*A*
	head–head*	C3*A*—O2*A*⋯C9*C*	146.0 (1)	3.541 (3)	146.6
					
10		C1*A*—O2*A*—C3*A*	106.52 (15)	341.5 (2)	
		C1*A*—O2*A*⋯C4*C*	135.3 (1)	pyramidal	pivot-O2*A*
	head–head*	C3*A*—O2*A*⋯C4*C*	99.7 (1)	3.535 (3)	169.1
					
11		C1*B*—O2*B*—C3*B*	106.77 (15)	334.1 (2)	singlet-O2*B*
		C1*B*—O2*B*⋯C11*C*	136.4 (1)	pyramidal	(146.7)*^*c*^*
	tail–head*	C3*B*—O2*B*⋯C11*C*	90.9 (1)	3.372 (3)	176.9
					
12		C1*A*—O1*A*—C2*A*	107.92 (17)	354.2 (2)	
		C1*A*—O1*A*⋯C7*B*	111.1 (1)	planar	singlet-O1*A*
	head–head*	C2*A*—O1*A*⋯C7*B*	135.2 (1)	3.261 (2)	165.8
					
13		C1*B*—O1*B*—C2*B*	107.75 (16)	356.1 (2)	
		C1*B*—O1*B*⋯C7*A*	107.6 (1)	planar	couplet-7
	head–head*	C2*B*—O1*B*⋯C7*A*	140.7 (1)	3.393 (2)	142.5
					
14		C1*C*—O1*C*—C2*C*	107.30 (16)	352.9 (2)	
		C1*C*—O1*C*⋯C16*B*	147.7 (1)	planar	couplet-11
	tail–head*	C2*C*—O1*C*⋯C16*B*	97.9 (1)	3.584 (3)	149.1

Notes: (*a*) atoms O2*C*, O3*B*, O4*A*–O4*C* and O5*B* showed no close contacts. (*b*) Configuration assigned arbitrarily based on the magnitude of the angle sum: pyramidal <344° and planar >344°. (*c*) The C11*C*—H11*C*⋯C3*B* angle.
