# Supplementary material for: 3,4-Bis-O-propargyl-1,2:5,6-di-O-iso­propyl­idene-d-mannitol: a study of multiple weak hydrogen bonds in the solid state
Source: Acta Crystallogr C Struct Chem. 2022 Oct 11;78(Pt 11):629–46. doi: 10.1107/S205322962200897X (PMC9635589; doi:10.1107/S205322962200897X)
Supplement: Supplementary file 4 [file c-78-00629-sup4.pdf]

STRUCTURAL CHEMISTRY

Volume 78 (2022)

Supporting information for article:

3,4-Bis-*O*-propargyl-1,2:5,6-di-*O*-isopropylidene-d-mannitol: a study of multiple weak hydrogen bonds in the solid state

Adnan I. Mohammed, Mohan M. Bhadbhade and Roger W. Read

Table of contents

S1. Experimental

S1.1. Synthesis

S1. S1.2. Refinement

Table S1 Table S1. Hydrogen-bond geometry.

1.1. S1.3. Analyses of the Cambridge Structural Database (CSD)

1.1.1. S1.3.1. Searches of the CSD based on MERCURY Crystal Packing Features (PFF)

Figure S1 Figure S1. MERCURY Crystal Packing Features (PFF) used for searches of the CSD.

Figure S2 Figure S2. Structural features of 1 used to define PFF and CSM contact search motifs.

Table S2 Table S2. Structure codes of positive and negative hits derived from MERCURY PFF searches.

S2. S1.3.2. Searches based on liberally defined structural motifs using the CONQUEST search tool

Figure S3 Figure S3. CONQUEST search motifs and CSD search results for Index Numbers *versus* D1, D2 (Å) and ANG (deg).

Figure 1 Figure S4. Molecular structures of compounds with outlier contacts in Figure 10(*c*).

S2. References

S1. Experimental

S1.1. Synthesis

The synthesis of 3,4-bis-*O*-propargyl-1,2:5,6-di-*O*-isopropylidene-D-mannitol **1** (IUPAC systematic name: (1*R*,2*R*)-1,2-bis((*R*)-2,2-dimethyl-1,3-dioxolan-4-yl)-1,2-bis(prop-2-yn-1-yloxy)ethane has been reported (Mohammed, 2012) and the X-ray diffraction sample crystallized from EtOAc/*n*-hexane as colourless prisms, m.p. 50-52 °C.

S1.2. Refinement

A colourless block like crystal of **1** with dimensions of 0.19 X 0.21 X 0.24 mm^3, selected under the polarizing microscope (Leica M165Z), was picked up on a MicroMount (MiTeGen, USA) consisting of a thin polymer tip with a wicking aperture. The X-ray diffraction measurements were carried out on a Bruker kappa-II CCD diffractometer at 150 K using IμS Incoatec Microfocus Source with Mo-Kα radiation (λ = 0.710723 Å). The single crystal, mounted on the goniometer using a cryo loop for intensity measurements, was coated with immersion oil type NVH and then quickly transferred to the cold nitrogen stream generated by an Oxford Cryostream 700 series. Symmetry related absorption corrections using the program SADABS (Bruker, 2016) were applied and the data were corrected for Lorentz and polarisation effects using Bruker APEX3 software (Bruker, 2016). The structure was solved by program SHELXT (Sheldrick, 2015*a*) (with intrinsic phasing) and the full-matrix least-square refinements were carried out using SHELXL (Sheldrick, 2015*b*) through the OLEX2 (Dolomanov, 2009) suite of software. Details of the experimental crystallographic data collected for compound **1** are summarized in Table 1. The non-hydrogen atoms were refined anisotropically but the hydrogen atoms were not located in the difference Fourier map. Instead, hydrogen atoms were geometrically placed and constrained according to their environment using different AFIX commands available in SHELXL (Sheldrick, 2015*b*) operating via the OLEX2 (Dolomanov, 2009) platform. The CCDC submission number is 2055328.

The geometric characteristics of hydrogen bonds are summarized in Table S1.

Table S1 Hydrogen-bond geometry (Å, °)

D—H···*A*	*D*—H	H···*A*	*D*···*A*	*D*—H···*A*
C7A—H7AA···O1B^{i}	0.99	2.55	3.393 (3)	142.5
C9A—H9A···O6C^{ii}	0.95	2.58	3.375 (3)	140.8

C13A—H13A···O6A^{iii}	0.95	2.34	3.246 (3)	158.2
C18A—H18B···O5C^{iv}	0.98	2.62	3.581 (3)	168.1
C5B—H5BA···O3A^{v}	0.98	2.59	3.525 (3)	160.0
C9B—H9B···O3C	0.95	2.19	3.121 (3)	167.1
C13B—H13B···O6B^{iii}	0.95	2.21	3.138 (3)	164.7
C16B—H16B···O1C	1.00	2.69	3.584 (2)	149.1
C4C—H4CB···O2A^{vi}	0.98	2.57	3.536 (3)	169.1
C6C—H6C···O5A^{vi}	1.00	2.67	3.458 (2)	135.7
C9C—H9C···O2A^{vii}	0.95	2.71	3.542 (3)	146.6
C11C—H11E···O2B^{viii}	0.99	2.38	3.372 (3)	176.9
C13C—H13C···O6C^{ix}	0.95	2.34	3.278 (3)	167.8

Symmetry codes: (i) *x*−1, *y*−1, *z*; (ii) *x*, *y*−1, *z*−1; (iii) *x*+1, *y*, *z*; (iv) *x*, *y*, *z*−1; (v) *x*+1, *y*+1, *z*; (vi) *x*, *y*, *z*+1; (vii) *x*, *y*+1, *z*+1; (viii) *x*−1, *y*, *z*; (ix) *x*, *y*−1, *z*.

S1.3. Analyses of the Cambridge Structural Database (CSD)

S1.3.1. Searches of the CSD based on MERCURY Crystal Packing Features (PFF)

A total of thirty-three individual searches of the Cambridge Structural Database (CSD) for Crystal Packing Features (referred to here as PFFs) illustrated in Figure S1 were carried out on eleven unique sets of Donor ***D*** (A1, A2, B, C, D) and Acceptor ***A*** (E, F, G, H, I, J) propargylic contacts that were recognised within the crystal structure of compound **1** (Fig. S2). Search criteria specified consideration of Cyclicity and were given a Low setting tolerance Level of Geometric Similarity. Where bifurcation was evident, individual PFF searches were performed for each partner pair and then for the two interactions together. The output of each search was recorded with a Positive result (a numerical and itemized list of known structures, with structure codes, that fell within the Low Level of Geometric Similarity), and a Negative result (included a corresponding numerical and itemized list of known structures containing the components of the search query, but where the geometric tolerances were not met). The reference codes of structures regarded as Positive and

Negative hits under each PFF search result, and their total numbers and percentages, were compiled into Microsoft Excel spreadsheets. A spreadsheet of the results with matching Positive and Negative structure codes aligned (with the exception of search B1.2) was constructed (Table S2), and the numerical data summarized in graphical form (Fig. 9, see Section 3.4.2).

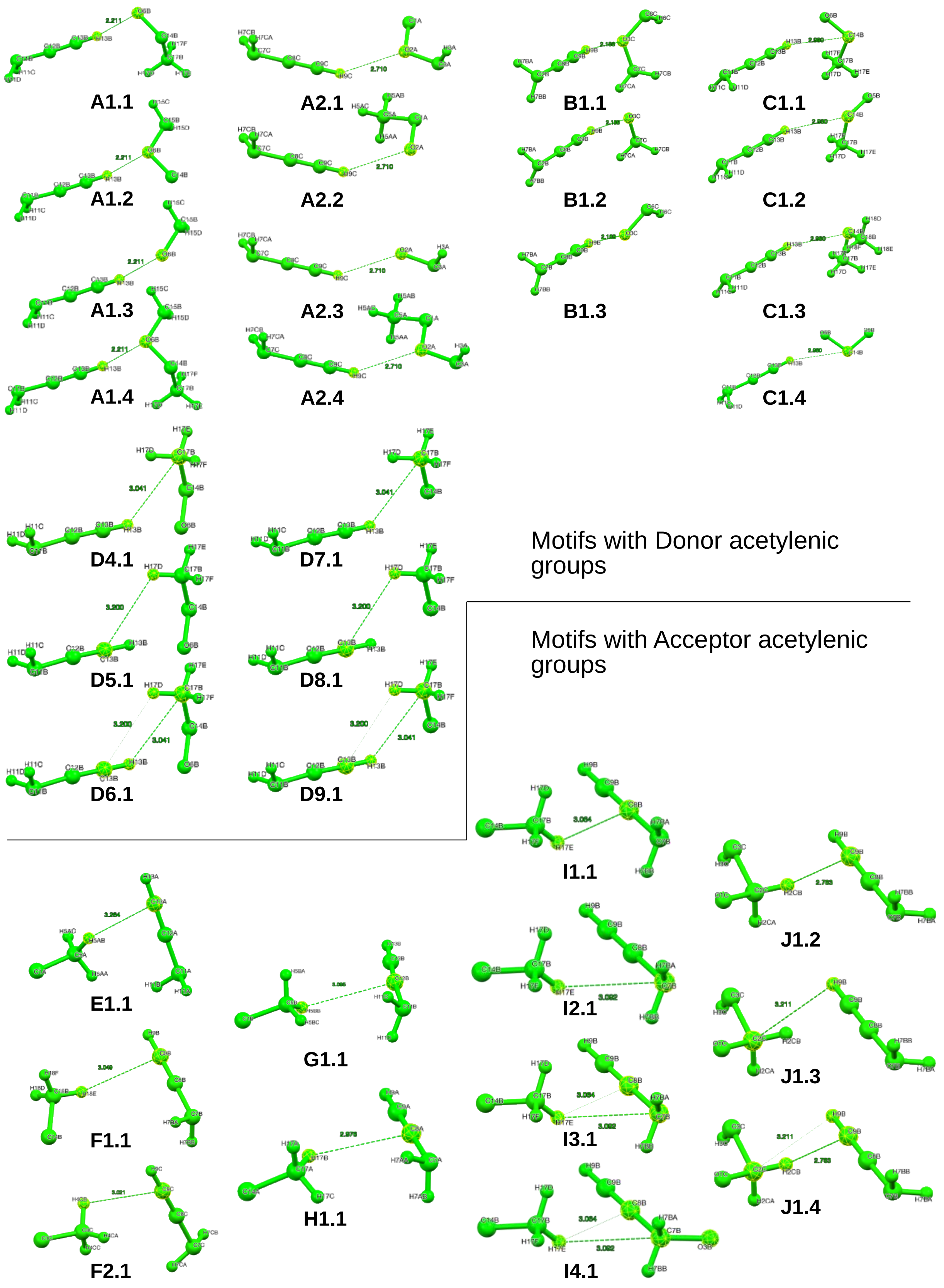

Figure S1 MERCURY Crystal Packing Features (PFF) used for searches of the CSD.

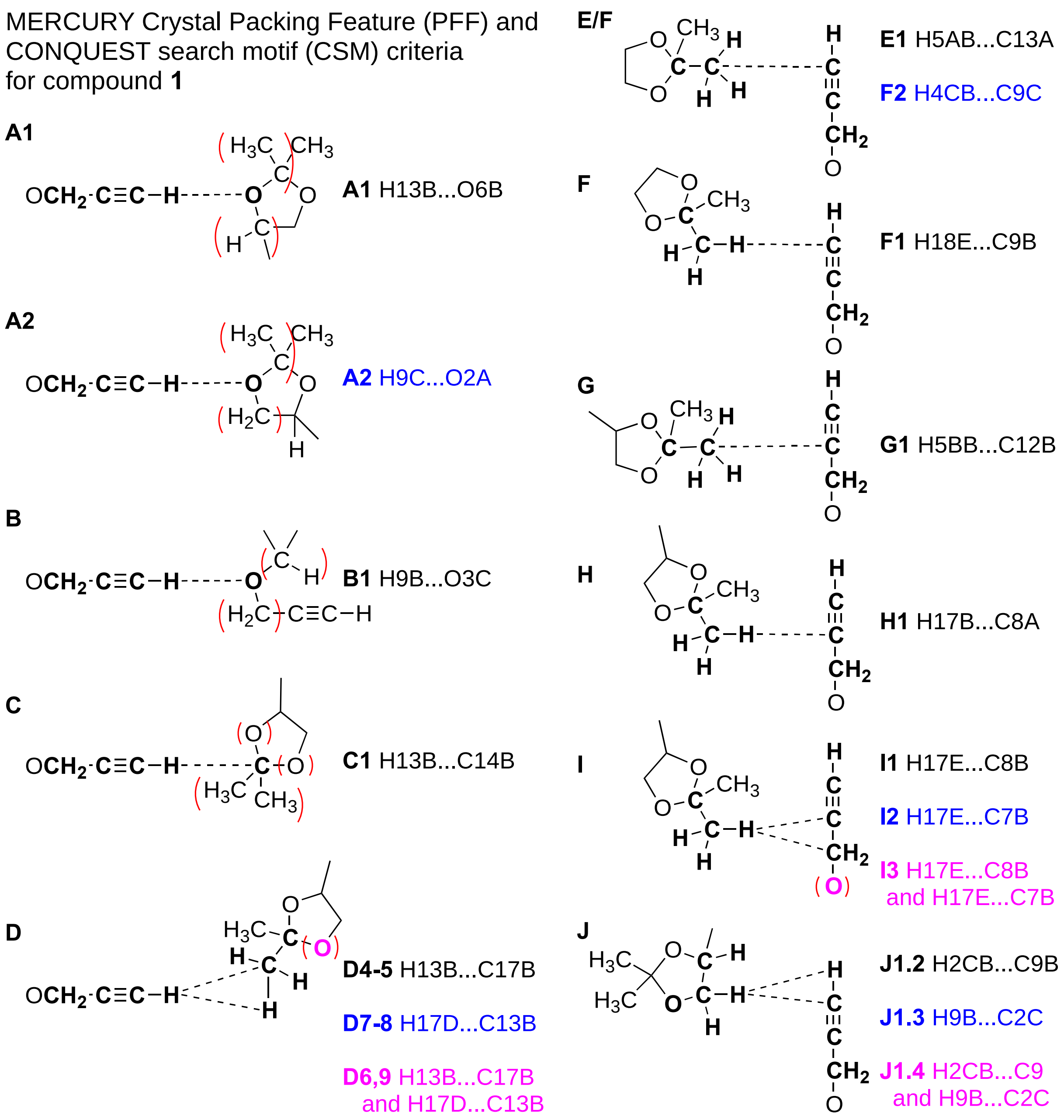

NOTES: (1) ***D***...***A*** elements are as indicated after the search code; (2) **Fixed** search elements are indicated in bold; (3) Individually **varied** search elements are marked in parentheses.

Figure S2 Structural features of **1** used to define PFF and CSM contact search motifs.

Table S2 Structure codes of positive and negative hits derived from MERCURY PFF searches (Table contains two parts).

Search	A1.1	A1.2	A1.3	A1.4	A2.1	A2.2	A2.3	A2.4	B1.1	B1.2	B1.3	C1.1	C1.2	C1.3	C1.4	D4	D5	D6	D7	D8	D9	E1	F1	F2	G1	H1	I1	I2	I3	I4	J1.2	J1.3	J1.4
Positive hits	1	1	1	0	5	7	4	2	2	6	5	9	4	5	6	8	8	8	10	10	10	0	4	3	1	3	2	2	2	0	0	0	0
Negative hits	-9	-9	-34	-5	-20	-21	-61	-10	-5	-330	-17	-19	-24	-29	-15	-20	-20	-20	-66	-66	-66	-71	-72	-73	-75	-73	-74	-74	-74	-27	-11	-11	-11
Total hits	10	10	35	5	25	28	65	12	7	336	22	28	28	34	21	28	28	28	76	76	76	71	76	76	76	76	76	76	76	27	11	11	11
% Positive	10	10	2.9	0	20	25	6.2	16.7	28.6	1.8	22.7	32.1	14.3	14.7	28.6	28.6	28.6	28.6	13.2	13.2	13.2	0	5.3	3.9	1.3	3.9	2.6	2.6	2.6	0	0	0	0
% Negative	90	90	97.1	100	80	75	93.8	83.3	71.4	98.2	77.3	67.9	85.7	85.3	71.4	71.4	71.4	71.4	86.8	86.8	86.8	100	94.7	96.1	98.7	96.1	97.4	97.4	97.4	100	100	100	100
**Pos RefCodes**																																	
						BAGWAJ										BAGWAJ	BAGWAJ	BAGWAJ															
					BEQGAI							BEQGAI	BEQGAI	BEQGAI	BEQGAI	BEQGAI	BEQGAI	BEQGAI	BEQGAI	BEQGAI	BEQGAI	–											
																							EHAKAZ										
										EMAQEQ																							
					EQAFEJ										EQAFEJ								EQAFEJ										
											FEHNUD																						
									GINGAM	GINGAM																							
											GOYZIG																						
							HAMRUJ																										
						HIHJEQ		HIHJEQ				HIHJEQ				HIHJEQ	HIHJEQ	HIHJEQ	HIHJEQ	HIHJEQ	HIHJEQ												
																								IHAFOO									
							JEBQIT																										
	JEWQIM	JEWQIM	JEWQIM												JEWQIM																		
										KEVWOZ																							
									KELSOL		KELSOL																						
										KUPNEO																							
					KUPNER	KUPNER						KUPNER																					
														KUPNUH		KUPNUH	KUPNUH	KUPNUH															
						MAYKAB						MAYKAB	MAYKAB	MAYKAB	MAYKAB	MAYKAB	MAYKAB	MAYKAB	MAYKAB	MAYKAB	MAYKAB												
																			MOKBOF	MOKBOF	MOKBOF												
					MUBBEQ		MUBBEQ																										
												NEPXUC				NEPXUC	NEPXUC	NEPXUC	NEPXUC	NEPXUC	NEPXUC												
																								OXULEA									
											PEQKON																						
																			QUYTIP	QUYTIP	QUYTIP												
																											SAXLIP	SAXLIP	SAXLIP				
						SOQBEG						SOQBEG				SOQBEG	SOQBEG	SOQBEG	SOQBEG	SOQBEG	SOQBEG												
													SOQBIK										SOQBIK										
						SUTWAI						SUTWAI			SUTWAI								SUTWAI										
												VOPHIU		VOPHIU		VOPHIU	VOPHIU	VOPHIU	VOPHIU	VOPHIU	VOPHIU												
											WALQUV																						
										WOWMIH																							
					WUJWAC	WUJWAC	WUJWAC	WUJWAC				WUJWAC			WUJWAC																		
													XIVCIO	XIVCIO					XIVCIO	XIVCIO	XIVCIO												
										XOXDOG																							
																			YAHLUQ	YAHLUQ	YAHLUQ						YAHLUQ	YAHLUQ	YAHLUQ				
																								YITFOY		YITFOY							
																										ZEXMIY							
																									ZEXMOE	ZEXMOE							
**Neg RefCodes**										330 Neg																							
							ACEHEW																										
							ACEROQ																										
			ASUHOM																														
												BAGWAJ	BAGWAJ						BAGWAJ	BAGWAJ	BAGWAJ	BAGWAJ	BAGWAJ	BAGWAJ	BAGWAJ	BAGWAJ	BAGWAJ	BAGWAJ	BAGWAJ	BAGWAJ			
						BEQGAI	BEQGAI	BEQGAI														BEQGAI	BEQGAI	BEQGAI	BEQGAI	BEQGAI	BEQGAI	BEQGAI	BEQGAI	BEQGAI			
					BOCFOO		BOCFOO				BOCFOO																						
																			CEKGEH	CEKGEH	CEKGEH	CEKGEH	CEKGEH	CEKGEH	CEKGEH	CEKGEH	CEKGEH	CEKGEH	CEKGEH				
							CIZFAV												CIZFAV	CIZFAV	CIZFAV	CIZFAV	CIZFAV	CIZFAV	CIZFAV	CIZFAV	CIZFAV	CIZFAV	CIZFAV	CIZFAV			
											COSRAE																						
									DIFQOZ		DIFQOZ																						
							EFAWAL																										
																			EFEQIR	EFEQIR	EFEQIR	EFEQIR	EFEQIR	EFEQIR	EFEQIR	EFEQIR	EFEQIR	EFEQIR	EFEQIR				
	EHAKAZ	EHAKAZ	EHAKAZ	EHAKAZ	EHAKAZ	EHAKAZ	EHAKAZ	EHAKAZ				EHAKAZ	EHAKAZ	EHAKAZ	EHAKAZ	EHAKAZ	EHAKAZ	EHAKAZ	EHAKAZ	EHAKAZ	EHAKAZ	EHAKAZ		EHAKAZ	EHAKAZ	EHAKAZ	EHAKAZ	EHAKAZ	EHAKAZ		EHAKAZ	EHAKAZ	EHAKAZ
	EMAKIN	EMAKIN	EMAKIN											EMAKIN	EMAKIN				EMAKIN	EMAKIN	EMAKIN	EMAKIN	EMAKIN	EMAKIN	EMAKIN	EMAKIN	EMAKIN	EMAKIN	EMAKIN				
							ENIJIV																										
			EQAFAF		EQAFAF	EQAFAF	EQAFAF					EQAFAF	EQAFAF	EQAFAF	EQAFAF	EQAFAF	EQAFAF	EQAFAF	EQAFAF	EQAFAF	EQAFAF	EQAFAF	EQAFAF	EQAFAF	EQAFAF	EQAFAF	EQAFAF	EQAFAF	EQAFAF	EQAFAF			
			EQAFEJ			EQAFEJ	EQAFEJ					EQAFEJ	EQAFEJ	EQAFEJ		EQAFEJ	EQAFEJ	EQAFEJ	EQAFEJ	EQAFEJ	EQAFEJ	EQAFEJ		EQAFEJ	EQAFEJ	EQAFEJ	EQAFEJ	EQAFEJ	EQAFEJ	EQAFEJ			
							ESUQOZ																										
							ETIROQ																										
			FAKZID				FAKZID																								FAKZID	FAKZID	FAKZID
							FOMJID																										
											GINGAM																						
									GURDAB		GURDAB																						
							GUSYUQ																										
																			HALSUJ	HALSUJ	HALSUJ	HALSUJ	HALSUJ	HALSUJ	HALSUJ	HALSUJ	HALSUJ	HALSUJ	HALSUJ				
					HIHJEQ		HIHJEQ						HIHJEQ									HIHJEQ	HIHJEQ	HIHJEQ	HIHJEQ	HIHJEQ	HIHJEQ	HIHJEQ	HIHJEQ	HIHJEQ			
											HIRJEZ			HIRJEZ					HIRJEZ	HIRJEZ	HIRJEZ	HIRJEZ	HIRJEZ	HIRJEZ	HIRJEZ	HIRJEZ	HIRJEZ	HIRJEZ	HIRJEZ	HIRJEZ			
											HIRJEZ01			HIRJEZ01					HIRJEZ01	HIRJEZ01	HIRJEZ01	HIRJEZ01	HIRJEZ01	HIRJEZ01	HIRJEZ01	HIRJEZ01	HIRJEZ01	HIRJEZ01	HIRJEZ01	HIRJEZ01			
																			HITKIG	HITKIG	HITKIG	HITKIG	HITKIG	HITKIG	HITKIG	HITKIG	HITKIG	HITKIG	HITKIG				
			HURJEL																														
			HURKIQ				HURKIQ																										
																			HURLEN	HURLEN	HURLEN	HURLEN	HURLEN	HURLEN	HURLEN	HURLEN	HURLEN	HURLEN	HURLEN				
							HUVGIP																										
							HUVGOV																										
							HUVGUB																										
														IGAZOF					IGAZOF	IGAZOF	IGAZOF	IGAZOF	IGAZOF	IGAZOF	IGAZOF	IGAZOF	IGAZOF	IGAZOF	IGAZOF	IGAZOF			
	IHAFOO	IHAFOO	IHAFOO	IHAFOO	IHAFOO	IHAFOO	IHAFOO	IHAFOO				IHAFOO	IHAFOO	IHAFOO	IHAFOO	IHAFOO	IHAFOO	IHAFOO	IHAFOO	IHAFOO	IHAFOO	IHAFOO	IHAFOO		IHAFOO	IHAFOO	IHAFOO	IHAFOO	IHAFOO		IHAFOO	IHAFOO	IHAFOO
			IYEYES																														
							JEVVEO																										
																			JUBTOS	JUBTOS	JUBTOS		JUBTOS	JUBTOS	JUBTOS	JUBTOS	JUBTOS	JUBTOS	JUBTOS				
																			KELSOL	KELSOL	KELSOL	KELSOL	KELSOL	KELSOL	KELSOL	KELSOL	KELSOL	KELSOL	KELSOL	KELSOL			
			KEWYIX																														
	KIJDUD	KIJDUD	KIJDUD	KIJDUD		KIJDUD						KIJDUD	KIJDUD		KIJDUD	KIJDUD	KIJDUD	KIJDUD	KIJDUD	KIJDUD	KIJDUD	KIJDUD	KIJDUD	KIJDUD	KIJDUD	KIJDUD	KIJDUD	KIJDUD	KIJDUD				
							KOVQAP																										
	KUPNER	KUPNER	KUPNER				KUPNER	KUPNER					KUPNER	KUPNER	KUPNER	KUPNER	KUPNER	KUPNER	KUPNER	KUPNER	KUPNER		KUPNER	KUPNER	KUPNER	KUPNER	KUPNER	KUPNER	KUPNER		KUPNER	KUPNER	KUPNER
	KUPNUH	KUPNUH	KUPNUH	KUPNUH		KUPNUH						KUPNUH	KUPNUH		KUPNUH				KUPNUH	KUPNUH	KUPNUH		KUPNUH	KUPNUH	KUPNUH	KUPNUH	KUPNUH	KUPNUH	KUPNUH		KUPNUH	KUPNUH	KUPNUH
			KUWZOS																														
						LENHOE						LENHOE	LENHOE	LENHOE		LENHOE	LENHOE	LENHOE	LENHOE	LENHOE	LENHOE	LENHOE	LENHOE	LENHOE	LENHOE	LENHOE	LENHOE	LENHOE	LENHOE				
			LIHHIV																												LIHHIV	LIHHIV	LIHHIV

Search	A1.1	A1.2	A1.3	A1.4	A2.1	A2.2	A2.3	A2.4	B1.1	B1.2	B1.3	C1.1	C1.2	C1.3	C1.4	D4	D5	D6	D7	D8	D9	E1	F1	F2	G1	H1	I1	I2
Positive hits	1	1	1	0	5	7	4	2	2	6	5	9	4	5	6	8	8	8	10	10	10	0	4	3	1	3	2	[illegible]
Negative hits	-9	-9	-34	-5	-20	-21	-61	-10	-5	-330	-17	-19	-24	-29	-15	-20	-20	-20	-66	-66	-66	-71	-72	-73	-75	-73	-74	[illegible]
Total hits	10	10	35	5	25	28	65	12	7	336	22	28	28	34	21	28	28	28	76	76	76	71	76	76	76	76	76	[illegible]
														LUYLOI					LUYLOI	LUYLOI	LUYLOI	LUYLOI	LUYLOI	LUYLOI	LUYLOI	LUYLOI	LUYLOI	[illegible]
					MAYKAB		MAYKAB	MAYKAB														MAYKAB	MAYKAB	MAYKAB	MAYKAB	MAYKAB	MAYKAB	[illegible]
																			MILLEY	MILLEY	MILLEY	MILLEY	MILLEY	MILLEY	MILLEY	MILLEY	MILLEY	[illegible]
														MOKBOF								MOKBOF	MOKBOF	MOKBOF	MOKBOF	MOKBOF	MOKBOF	[illegible]
						MUMQER	MUMQER					MUMQER	MUMQER			MUMQER	MUMQER	MUMQER	MUMQER	MUMQER	MUMQER	MUMQER	MUMQER	MUMQER	MUMQER	MUMQER	MUMQER	[illegible]
					NEPXUC	NEPXUC	NEPXUC	NEPXUC					NEPXUC	NEPXUC								NEPXUC	NEPXUC	NEPXUC	NEPXUC	NEPXUC	NEPXUC	[illegible]
			NEQSEJ																									
														NICQEY					NICQEY	NICQEY	NICQEY	NICQEY	NICQEY	NICQEY	NICQEY	NICQEY	NICQEY	[illegible]
																			NIZMIS	NIZMIS	NIZMIS	NIZMIS	NIZMIS	NIZMIS	NIZMIS	NIZMIS	NIZMIS	[illegible]
			NODBEP																									
			NUDHOJ																									
			NUPPUL																									
									NUPVAX		NUPVAX																	
			OBUQAF																									
							OCACIG																					
							OCACIG01																					
	OJAJOA	OJAJOA	OJAJOA																									
											OLAVED																	
																			ORIDAW	ORIDAW	ORIDAW	ORIDAW	ORIDAW	ORIDAW	ORIDAW	ORIDAW	ORIDAW	[illegible]
			OXOZAD			OXOZAD						OXOZAD	OXOZAD			OXOZAD	OXOZAD	OXOZAD	OXOZAD	OXOZAD	OXOZAD	OXOZAD	OXOZAD	OXOZAD	OXOZAD	OXOZAD	OXOZAD	[illegible]
			OXULEA																OXULEA	OXULEA	OXULEA	OXULEA	OXULEA		OXULEA	OXULEA	OXULEA	[illegible]
			PAHJEO																									
							PALMEX																					
									PEQKON																			
																			PIHPOO	PIHPOO	PIHPOO	PIHPOO	PIHPOO	PIHPOO	PIHPOO	PIHPOO	PIHPOO	[illegible]
											PIJTEJ								PIJTEJ	PIJTEJ	PIJTEJ	PIJTEJ	PIJTEJ	PIJTEJ	PIJTEJ	PIJTEJ	PIJTEJ	[illegible]
							PRYURD10																					
			QAMTED				QAMTED																					
																			QIMGUQ	QIMGUQ	QIMGUQ	QIMGUQ	QIMGUQ	QIMGUQ	QIMGUQ	QIMGUQ	QIMGUQ	[illegible]
							QIPHII																					
														QOYSED					QOYSED	QOYSED	QOYSED	QOYSED	QOYSED	QOYSED	QOYSED	QOYSED	QOYSED	[illegible]
					QULLUG		QULLUG																					
			QUMZEF				QUMZEF																					
														QUYTEL					QUYTEL	QUYTEL	QUYTEL	QUYTEL	QUYTEL	QUYTEL	QUYTEL	QUYTEL	QUYTEL	[illegible]
														QUYTIP								QUYTIP	QUYTIP	QUYTIP	QUYTIP	QUYTIP	QUYTIP	[illegible]
							REGHUG												REGHUG	REGHUG	REGHUG	REGHUG	REGHUG	REGHUG	REGHUG	REGHUG	REGHUG	[illegible]
											RIRNUC																	
							RIWNEQ																					
					RODFIB		RODFIB																					
																			RODFUN	RODFUN	RODFUN	RODFUN	RODFUN	RODFUN	RODFUN	RODFUN	RODFUN	[illegible]
							ROHHII																					
					ROJVIY	ROJVIY	ROJVIY					ROJVIY	ROJVIY		ROJVIY	ROJVIY	ROJVIY	ROJVIY	ROJVIY	ROJVIY	ROJVIY	ROJVIY	ROJVIY	ROJVIY	ROJVIY	ROJVIY	ROJVIY	[illegible]
					ROJVOE	ROJVOE	ROJVOE					ROJVOE	ROJVOE		ROJVOE	ROJVOE	ROJVOE	ROJVOE	ROJVOE	ROJVOE	ROJVOE	ROJVOE	ROJVOE	ROJVOE	ROJVOE	ROJVOE	ROJVOE	[illegible]
																			RUJZIH	RUJZIH	RUJZIH	RUJZIH	RUJZIH	RUJZIH	RUJZIH	RUJZIH	RUJZIH	[illegible]
							SASFID												SASFID	SASFID	SASFID	SASFID	SASFID	SASFID	SASFID	SASFID	SASFID	[illegible]
							SAXLIP							SAXLIP					SAXLIP	SAXLIP	SAXLIP	SAXLIP	SAXLIP	SAXLIP	SAXLIP	SAXLIP		
																			SESDAX	SESDAX	SESDAX	SESDAX	SESDAX	SESDAX	SESDAX	SESDAX	SESDAX	[illegible]
													SOQBEG									SOQBEG	SOQBEG	SOQBEG	SOQBEG	SOQBEG	SOQBEG	[illegible]
						SOQBIK						SOQBIK			SOQBIK	SOQBIK	SOQBIK	SOQBIK	SOQBIK	SOQBIK	SOQBIK	SOQBIK		SOQBIK	SOQBIK	SOQBIK	SOQBIK	[illegible]
					SOQBOQ	SOQBOQ	SOQBOQ	SOQBOQ				SOQBOQ	SOQBOQ		SOQBOQ	SOQBOQ	SOQBOQ	SOQBOQ	SOQBOQ	SOQBOQ	SOQBOQ	SOQBOQ	SOQBOQ	SOQBOQ	SOQBOQ	SOQBOQ	SOQBOQ	[illegible]
																			SORKUI	SORKUI	SORKUI	SORKUI	SORKUI	SORKUI	SORKUI	SORKUI	SORKUI	[illegible]
							SOTPAU																					
	SUTWAI	SUTWAI	SUTWAI	SUTWAI	SUTWAI		SUTWAI	SUTWAI					SUTWAI	SUTWAI		SUTWAI	SUTWAI	SUTWAI	SUTWAI	SUTWAI	SUTWAI			SUTWAI	SUTWAI	SUTWAI	SUTWAI	[illegible]
																			TAQHIF	TAQHIF	TAQHIF	TAQHIF	TAQHIF	TAQHIF	TAQHIF	TAQHIF	TAQHIF	[illegible]
			TAXTOD																									
							TEHVAE																					
														TIKKUW					TIKKUW	TIKKUW	TIKKUW	TIKKUW	TIKKUW	TIKKUW	TIKKUW	TIKKUW	TIKKUW	[illegible]
														TOWJUM					TOWJUM	TOWJUM	TOWJUM	TOWJUM	TOWJUM	TOWJUM	TOWJUM	TOWJUM	TOWJUM	[illegible]
			UGEBUD																									
																			UVASEQ	UVASEQ	UVASEQ	UVASEQ	UVASEQ	UVASEQ	UVASEQ	UVASEQ	UVASEQ	[illegible]
																			UVASIU	UVASIU	UVASIU	UVASIU	UVASIU	UVASIU	UVASIU	UVASIU	UVASIU	[illegible]
																			UVASOA	UVASOA	UVASOA	UVASOA	UVASOA	UVASOA	UVASOA	UVASOA	UVASOA	[illegible]
							UXAYEY																					
														UXOGAQ					UXOGAQ	UXOGAQ	UXOGAQ	UXOGAQ	UXOGAQ	UXOGAQ	UXOGAQ	UXOGAQ	UXOGAQ	[illegible]
							VALVOW																					
																			VECDUF	VECDUF	VECDUF	VECDUF	VECDUF	VECDUF	VECDUF	VECDUF	VECDUF	[illegible]
			VIJKOP				VIJKOP																					
						VOPHIU							VOPHIU									VOPHIU	VOPHIU	VOPHIU	VOPHIU	VOPHIU	VOPHIU	[illegible]
					VOXQEG	VOXQEG	VOXQEG					VOXQEG	VOXQEG		VOXQEG	VOXQEG	VOXQEG	VOXQEG	VOXQEG	VOXQEG	VOXQEG	VOXQEG	VOXQEG	VOXQEG	VOXQEG	VOXQEG	VOXQEG	[illegible]
					VUBRUI		VUBRUI				VUBRUI																	
											VUDROE																	
					VUKZEH		VUKZEH																					
									WALQUV																			
																			WERPOB	WERPOB	WERPOB	WERPOB	WERPOB	WERPOB	WERPOB	WERPOB	WERPOB	[illegible]
																			WERPUH	WERPUH	WERPUH	WERPUH	WERPUH	WERPUH	WERPUH	WERPUH	WERPUH	[illegible]
							WIYXOU												WIYXOU	WIYXOU	WIYXOU	WIYXOU	WIYXOU	WIYXOU	WIYXOU	WIYXOU	WIYXOU	[illegible]
							WOYWOZ																					
													WUJWAC	WUJWAC		WUJWAC	WUJWAC	WUJWAC	WUJWAC	WUJWAC	WUJWAC		WUJWAC	WUJWAC	WUJWAC	WUJWAC	WUJWAC	[illegible]
							WUSQUY																					
			XESSIX																									
			XIVCIO		XIVCIO	XIVCIO	XIVCIO	XIVCIO				XIVCIO			XIVCIO	XIVCIO	XIVCIO	XIVCIO				XIVCIO	XIVCIO	XIVCIO	XIVCIO	XIVCIO	XIVCIO	[illegible]
											XOCTEP																	
					XOGHIL	XOGHIL	XOGHIL	XOGHIL			XOGHIL	XOGHIL	XOGHIL	XOGHIL	XOGHIL	XOGHIL	XOGHIL	XOGHIL	XOGHIL	XOGHIL	XOGHIL	XOGHIL	XOGHIL	XOGHIL	XOGHIL	XOGHIL	XOGHIL	[illegible]
														YAHLUQ								YAHLUQ	YAHLUQ	YAHLUQ	YAHLUQ	YAHLUQ		
						YEBSIJ	YEBSIJ					YEBSIJ	YEBSIJ			YEBSIJ	YEBSIJ	YEBSIJ	YEBSIJ	YEBSIJ	YEBSIJ	YEBSIJ	YEBSIJ	YEBSIJ	YEBSIJ	YEBSIJ	YEBSIJ	[illegible]
					YIBMEE		YIBMEE																					
					YIBMEE01		YIBMEE01																					
											YIFLUV																	
	YITFOY	YITFOY	YITFOY											YITFOY	YITFOY				YITFOY	YITFOY	YITFOY	YITFOY	YITFOY		YITFOY		YITFOY	[illegible]
						YITWUW						YITWUW	YITWUW	YITWUW		YITWUW	YITWUW	YITWUW	YITWUW	YITWUW	YITWUW	YITWUW	YITWUW	YITWUW	YITWUW	YITWUW	YITWUW	[illegible]
											YOCKEJ																	
														ZEXMIY					ZEXMIY	ZEXMIY	ZEXMIY	ZEXMIY	ZEXMIY	ZEXMIY	ZEXMIY		ZEXMIY	[illegible]
																			ZEXMOE	ZEXMOE	ZEXMOE	ZEXMOE	ZEXMOE	ZEXMOE			ZEXMOE	[illegible]
														ZODCED					ZODCED	ZODCED	ZODCED	ZODCED	ZODCED	ZODCED	ZODCED	ZODCED	ZODCED	[illegible]

S1.3.2. Searches based on liberally defined structural motifs using the CONQUEST search tool

Loosely constrained structural motifs derived from those shown in Figure S2 were established in the CONQUEST^{TM} search tool for propargylic Donor interactions: CSM_A1, CSM_A2, CSM1_R1, CSM1-R2, CSM1_R3, CSM1_R4, and Acceptor interactions: CSM1_R5 and CSM1_R6 (Fig. S3). Relevant distance parameters, D1 (**H**…***A***, Å) and D2 (***D***…***A***, Å), were liberally defined as within the sum of van der Waals radii + 1.0 Å, and the angular measurements, ANG (*D*–H…*A*, deg), limited to within 60—180 degrees. Where multiple contacts were recorded for a single compound, sometimes within the same category,

these were included for completeness. The values for D1, D2, and ANG for all matching contacts found in the CSD were recorded and the results displayed as scatter plots against Identity Number in Figure S3 and discussed more fully in Section 3.4.3.

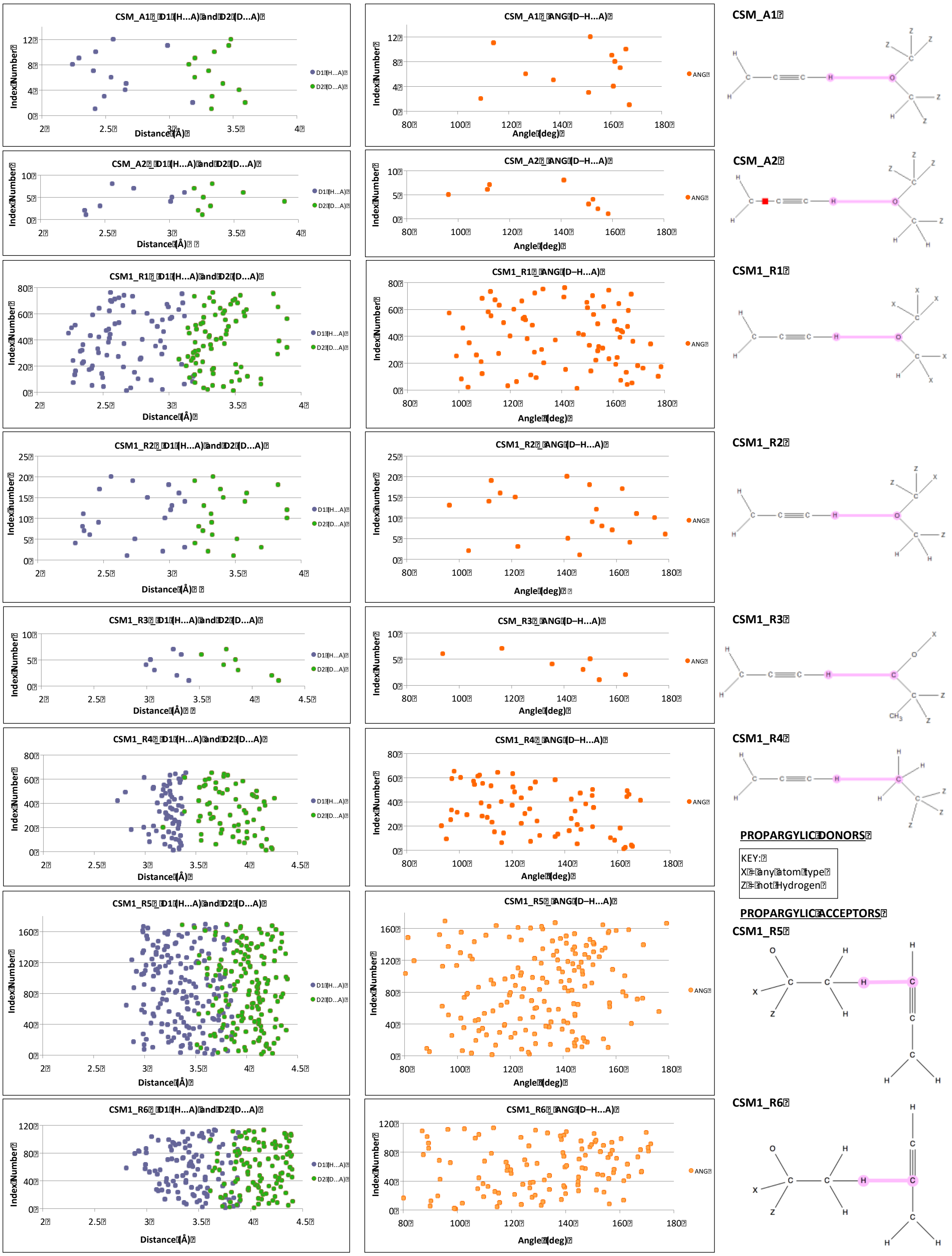

Figure S3 CONQUEST search motifs (right) and CSD search results showing scatter plots of Index Number versus D1 (blue) and D2 (green) (Å) (left) and ANG (orange) (deg) (middle) values.

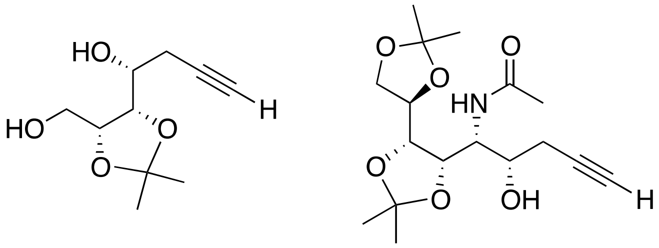

WUJWAC EHAKAZ

Figure S4 Molecular structures of compounds with outlier contacts in Figure 10(*c*).

S2. References

Bruker (2016). APEX3, SAINT and SADABS. Bruker AXS Inc., Madison, WI, USA.

Dolomanov, O. V., Bourhis, L. J., Gildea, R. J., Howard, J. A. K. & Puschmann, H. (2009). *J. Appl. Crystallogr.* **42**, 339–341.

Mohammed, A. I., Abboud, Z. H. & Alghanimi, A. H. O. (2012). *Tetrahedron Lett.* **53**, 5081–5083.

Sheldrick, G. M. (2015*a*). *Acta Cryst.* **A71**, 3–8. https://doi.org/10.1107/S2053273314026370

Sheldrick, G. M. (2015*b*). *Acta Cryst.* **C71**, 3–8. https://doi.org/10.1107/S2053229614024218
